# Rare variant analysis of 4241 pulmonary arterial hypertension cases from an international consortium implicates *FBLN2*, *PDGFD*, and rare de novo variants in PAH

**DOI:** 10.1186/s13073-021-00891-1

**Published:** 2021-05-10

**Authors:** Na Zhu, Emilia M. Swietlik, Carrie L. Welch, Michael W. Pauciulo, Jacob J. Hagen, Xueya Zhou, Yicheng Guo, Johannes Karten, Divya Pandya, Tobias Tilly, Katie A. Lutz, Jennifer M. Martin, Carmen M. Treacy, Erika B. Rosenzweig, Usha Krishnan, Anna W. Coleman, Claudia Gonzaga-Juaregui, Allan Lawrie, Richard C. Trembath, Martin R. Wilkins, Regeneron Genetics Center, PAH Biobank Enrolling Centers’ Investigators, Russel Hirsch, R. James White, Marc Simon, David Badesch, Erika Rosenzweig, Charles Burger, Murali Chakinala, Thenappan Thenappan, Greg Elliott, Robert Simms, Harrison Farber, Robert Frantz, Jean Elwing, Nicholas Hill, Dunbar Ivy, James Klinger, Steven Nathan, Ronald Oudiz, Ivan Robbins, Robert Schilz, Terry Fortin, Jeffrey Wilt, Delphine Yung, Eric Austin, Ferhaan Ahmad, Nitin Bhatt, Tim Lahm, Adaani Frost, Zeenat Safdar, Zia Rehman, Robert Walter, Fernando Torres, Sahil Bakshi, Stephen Archer, Rahul Argula, Christopher Barnett, Raymond Benza, Ankit Desai, Veeranna Maddipati, NIHR BioResource for Translational Research - Rare Diseases, Harm J. Bogaard, Colin Church, Gerry Coghlin, Robin Condliffe, Mélanie Eyries, Henning Gall, Stefano Ghio, Barbara Girerd, Simon Holden, Luke Howard, Marc Humbert, David G. Kiely, Gabor Kovacs, Jim Lordan, Rajiv D. Machado, Robert V. MacKenzie Ross, Colm McCabe, Jennifer M. Martin, Shahin Moledina, David Montani, Horst Olschewski, Christopher J. Penkett, Joanna Pepke-Zaba, Laura Price, Christopher J. Rhodes, Werner Seeger, Florent Soubrier, Laura Southgate, Jay Suntharalingam, Andrew J. Swift, Mark R. Toshner, Carmen M. Treacy, Anton Vonk Noordegraaf, John Wharton, Jim Wild, Stephen John Wort, National Cohort Study of Idiopathic and Heritable PAH, Harm J. Bogaard, Colin Church, Gerry Coghlin, Robin Condliffe, Mélanie Eyries, Henning Gall, Stefano Ghio, Barbara Girerd, Simon Holden, Luke Howard, Marc Humbert, David G. Kiely, Gabor Kovacs, Jim Lordan, Rajiv D. Machado, Robert V. MacKenzie Ross, Colm McCabe, Jennifer M. Martin, Shahin Moledina, David Montani, Horst Olschewski, Christopher J. Penkett, Joanna Pepke-Zaba, Laura Price, Christopher J. Rhodes, Werner Seeger, Florent Soubrier, Laura Southgate, Jay Suntharalingam, Andrew J. Swift, Mark R. Toshner, Carmen M. Treacy, Anton Vonk Noordegraaf, John Wharton, Jim Wild, Stephen John Wort, Nicholas W. Morrell, Yufeng Shen, Stefan Gräf, William C. Nichols, Wendy K. Chung

**Affiliations:** 1 Department of Pediatrics, Columbia University Irving Medical Center, 1150 St. Nicholas Avenue, Room 620, New York, NY 10032 USA; 2 Department of Systems Biology, Columbia University, New York, NY USA; 3 Department of Medicine, University of Cambridge, Cambridge Biomedical Campus, Cambridge, UK; 4 Division of Human Genetics, Cincinnati Children’s Hospital Medical Center, Cincinnati, OH USA; 5 Department of Pediatrics, University of Cincinnati College of Medicine, Cincinnati, OH USA; 6 42Genetics, Belfast, Ireland; 7 NIHR BioResource for Translational Research, Cambridge Biomedical Campus, Cambridge, UK; 8 Regeneron Pharmaceuticals, New York, NY USA; 9 Department of Infection, Immunity and Cardiovascular Disease, University of Sheffield, Sheffield, UK; 10 Department of Medical and Molecular Genetics, King’s College London, London, UK; 11 National Heart & Lung Institute, Imperial College London, London, UK; 12 www.pahbiobank.org, Cincinnati, OH USA; 13 University of Cambridge and Cambridge University Hospitals NHS Foundation Trust, Cambridge Biomedical Campus, Cambridge, UK; 14 www.ipahcohort.com, Cambridge, UK; 15 Addenbrooke’s Hospital NHS Foundation Trust, Cambridge Biomedical Campus, Cambridge, UK; 16 Royal Papworth Hospital NHS Foundation Trust, Cambridge Biomedical Campus, Cambridge, UK; 17 Department of Biomedical Informatics, Columbia University, New York, NY USA; 18 Department of Haematology, University of Cambridge, Cambridge Biomedical Campus, Cambridge, UK; 19 Herbert Irving Comprehensive Cancer Center, Columbia University Irving Medical Center, New York, NY USA; 20 Department of Medicine, Columbia University Irving Medical Center, New York, NY USA

**Keywords:** Genetics, Pulmonary arterial hypertension, Exome sequencing, Genome sequencing, Case-control association testing, De novo variant analysis

## Abstract

**Background:**

Pulmonary arterial hypertension (PAH) is a lethal vasculopathy characterized by pathogenic remodeling of pulmonary arterioles leading to increased pulmonary pressures, right ventricular hypertrophy, and heart failure. PAH can be associated with other diseases (APAH: connective tissue diseases, congenital heart disease, and others) but often the etiology is idiopathic (IPAH). Mutations in bone morphogenetic protein receptor 2 (*BMPR2*) are the cause of most heritable cases but the vast majority of other cases are genetically undefined.

**Methods:**

To identify new risk genes, we utilized an international consortium of 4241 PAH cases with exome or genome sequencing data from the National Biological Sample and Data Repository for PAH, Columbia University Irving Medical Center, and the UK NIHR BioResource – Rare Diseases Study. The strength of this combined cohort is a doubling of the number of IPAH cases compared to either national cohort alone. We identified protein-coding variants and performed rare variant association analyses in unrelated participants of European ancestry, including 1647 IPAH cases and 18,819 controls. We also analyzed de novo variants in 124 pediatric trios enriched for IPAH and APAH-CHD.

**Results:**

Seven genes with rare deleterious variants were associated with IPAH with false discovery rate smaller than 0.1: three known genes (*BMPR2*, *GDF2*, and *TBX4*), two recently identified candidate genes (*SOX17*, *KDR*), and two new candidate genes (fibulin 2, *FBLN2*; platelet-derived growth factor D, *PDGFD*). The new genes were identified based solely on rare deleterious missense variants, a variant type that could not be adequately assessed in either cohort alone. The candidate genes exhibit expression patterns in lung and heart similar to that of known PAH risk genes, and most variants occur in conserved protein domains. For pediatric PAH, predicted deleterious de novo variants exhibited a significant burden compared to the background mutation rate (2.45×, *p* = 2.5e−5). At least eight novel pediatric candidate genes carrying de novo variants have plausible roles in lung/heart development.

**Conclusions:**

Rare variant analysis of a large international consortium identified two new candidate genes—*FBLN2* and *PDGFD*. The new genes have known functions in vasculogenesis and remodeling. Trio analysis predicted that ~ 15% of pediatric IPAH may be explained by de novo variants.

**Supplementary Information:**

The online version contains supplementary material available at 10.1186/s13073-021-00891-1.

## Background

Pulmonary arterial hypertension (PAH) remains a progressive, lethal vasculopathy despite recent therapeutic advances. The disease is characterized by pulmonary vascular endothelial dysfunction and proliferative remodeling giving rise to increased pulmonary artery pressures and pulmonary vascular resistance. These pathological changes of the lung vasculature strain the right ventricle of the heart, leading to right ventricular hypertrophy, right heart failure, and high mortality [1–3]. Dysregulated vascular, inflammatory, and immune cells contribute to these pathological processes [3]. PAH can present at any age, but the ~ 3:1 female to male ratio in adult-onset disease is not observed in pediatric-onset disease, in which the disease incidence is similar for males and females. The estimated prevalence of PAH is 4.8–8.1 cases/million for pediatric-onset [4] and 5.6–25 cases/million for adult-onset disease [5]. Early genetic linkage and candidate gene studies indicated an autosomal dominant mode of inheritance for PAH risk. However, the known susceptibility variants are incompletely penetrant, many individuals who carry monogenic risk variants never develop PAH, and a subset of patients have deleterious variants in more than one risk gene. For example, bone morphogenetic protein receptor type 2 (*BMPR2*) mutations are observed in 60–80% of familial (FPAH) cases, but data from population registries indicate that penetrance of the disease phenotype ranges from 14 to 42% [6]. These data suggest that additional genetic, epigenetic, environmental factors, and gene × environment interactions contribute to disease.

Genetic analyses of larger cohorts using gene panels, exome sequencing (ES), or genome sequencing (GS) have further defined the frequency of individuals with deleterious variants in PAH risk genes and have identified novel candidate risk genes. *BMPR2* mutations are observed in the majority of FPAH cases across genetic ancestries [7–11]. *BMPR2* carriers have younger mean age-of-onset and are less responsive to vasodilators compared to non-carriers [7, 12, 13], with an enrichment of predicted deleterious missense (D-Mis) variants with younger age-of-onset [7, 14]. However, *BMPR2* variants have been identified in only 10–20% of previously classified idiopathic PAH (IPAH) and rarely to PAH associated with other diseases (APAH: autoimmune connective tissue diseases, congenital heart disease (CHD), portopulmonary disease and others) or PAH induced by diet and toxins. Variants in two other genes in the transforming growth factor-beta (TGF-β) superfamily, activin A receptor type II-like 1 (*ACVRL1*), and endoglin (*ENG*) contribute to ~ 0.8% of PAH cases [7], especially PAH associated with hereditary hemorrhagic telangiectasia (APAH-HHT). Variants in growth differentiation factor 2 (*GDF2*), encoding the ligand of BMPR2/ACVRL1 (BMP9), contribute to ~ 1% of PAH (mostly IPAH) cases in European-enriched cohorts [7, 8] and more frequently in Chinese patients (~ 6.7%) [15]. Variants in mothers against decapentaplegic (*SMAD*) genes, encoding downstream mediators of BMP signaling, contribute rarely.

A number of genes outside of the TGF-β signaling pathway have also been identified as PAH risk genes. Variants in developmental transcription factors, *TBX4* and *SOX17*, are enriched in pediatric patients [7, 16–18]. Each gene contributes to 7–8% of pediatric IPAH and ~ 5% (*TBX4*) or ~ 3.2% (*SOX17*) of pediatric APAH-CHD [19]. Originally described as a determinant of pattern formation including limb development [20], the association of *TBX4* with PAH, cardiac defects [21, 22], and a variety of developmental lung disorders [22, 23] indicate an expanding role for *TBX4* in embryonic development. Biallelic variants in eukaryotic initiation translation factor (*EIF2AK4*) cause pulmonary veno-occlusive disease (PVOD) and pulmonary capillary hemangiomatosis (PCH) [24, 25]. Loss of function variants in channelopathy genes potassium two pore domain channel (*KCNK3*) [26] and ATP-binding cassette subfamily member 8 (*ABCC8*) [27], as well as membrane reservoir gene caveolin-1 (*CAV1*) [28–30], are causative for PAH. Recent associations of variants in ATPase 13A3 (*ATP13A3*) and aquaporin 1 (*AQP1*) [8], as well as kallikrein 1 (*KLK1*) and gamma-glutamyl carboxylase (*GGCX*) [7], have been reported but require independent confirmation. Finally, a role for de novo variants in pediatric-onset PAH has been suggested based on a cohort of 34 child-parent trios [17].

Together, these data indicate that rare genetic variants underlie ~ 75–80% of FPAH [6], at least 10% of adult-onset idiopathic PAH (IPAH) [7, 8], and up to ~ 36% of pediatric-onset IPAH [31]. A substantial fraction of non-familial PAH cases remains genetically undefined. The low frequency of risk variants for each gene, except *BMPR2*, indicates that large numbers of individuals are required for further validation of rare risk genes and pathways, and to understand the natural history of each genetic subtype of PAH. Towards this end, we analyzed 4175 PAH cases from an international consortium with ES or GS. The National Biological Sample and Data Repository for PAH (aka PAH Biobank) was comprised of 2570 PAH cases (1110 IPAH and 1239 APAH) and the UK NIHR BioResource – Rare Diseases Study was comprised of 1144 cases, almost entirely IPAH. Thus, the increased power of the combined cohort was a 2-fold increase in the number of IPAH cases, and we focused our association analyses on this PAH subclass. The cohort size precluded testing of the oligogenicity hypothesis suggested by the incomplete penetrance of known PAH risk genes. Non-inherited de novo mutations could also contribute to genetically unexplained non-familial cases but require access to parental sequencing data. We previously showed that pediatric-onset PAH cases were enriched with damaging de novo variants. Here, we expand the analysis to a cohort of 124 pediatric child-parent trios.

## Methods

### Patient cohorts and control datasets

A total of 4175 PAH cases from the National Biological Sample and Data Repository for PAH (PAH Biobank, *n* = 2570 exomes) [7], UK NIHR BioResource – Rare Diseases Study (UK NIHR BioResource, *n* = 1144 genomes) [8], and the Columbia University Irving Medical Center (CUIMC, *n* = 461 exomes) [17, 18, 27] were included in a combined analysis of rare inherited variants. The subset of 124 affected child-unaffected parents trios (*n* = 111 CUIMC, *n* = 8 UK NIHR BioResource, *n* = 5 PAH Biobank) were included in an analysis of de novo variants. An additional 65 *BMPR2* mutation-positive cases from CUIMC without exome sequencing data were previously reported [17, 18] and included in the overall cohort counts (total of 4241 cases). As previously described, cases were diagnosed by medical record review including right heart catheterization and all were classified as World Symposium on Pulmonary Hypertension (WSPH) Group I [32]. Written informed consent for publication was obtained at enrollment. The studies were approved by the institutional review boards at CCHMC, individual PAH Biobank Centers, the East of England Cambridge South national research ethics committee (REC, ref. 13/EE0325) or CUIMC.

The control group consisted of unaffected parents from the Simons Powering Autism Research for Knowledge (SPARK) study (exomes) [33] as well as gnomADv2.1.1 (gnomAD) individuals (genomes).

### ES/GS data analysis

PAH Biobank, CUIMC, and SPARK cohort samples were all sequenced in collaboration with the Regeneron Genetics Center as previously described [7, 8, 17, 18, 27]; the UK NIHR BioResource sequence data were also previously described [8]. For case and SPARK control data, we used a previously established bioinformatics procedure [34] to process and analyze exome and genome sequence data. For the UK NIHR BioResource data, we extracted reads from GS data by the following procedure: (1) obtained all reads that were mapped to the human genome regions that overlapped with the target regions of xGEN exome capture intervals (Exome Research panel 1.0); (2) the mate pairs of these reads. We then processed the extracted GS data using the same pipeline as the ES data. Specifically, we used BWA-MEM [35] to map and align paired-end reads to the human reference genome (version GRCh38/hg38, accession GCA 000001405.15), Picard v1.93 MarkDuplicates to identify and flag PCR duplicates, and GATK v4.1 [36, 37] HaplotypeCaller in Reference Confidence Model mode to generate individual-level gVCF files from the aligned sequence data. We then performed joint calling of variants from all three datasets using *GLnexus* [38]. We used the following inclusion rules to select variants for downstream analysis: AF < 0.05% in the cohort, < 0.01% in gnomAD exome_ALL (all ancestries); > 90% target region with dp ≥ 10; mappability = 1; and allele balance ≥ 0.25. We also ran DeepVariants [39, 40], a new tool based on machine learning, for all cases and SPARK controls. We used the ES mode for ES data and GS mode for GS data, and then filtered by “PASS” DeepVariants. Inclusion criteria for variants observed in multiple carriers was ≥ 50% of all calls PASS DeepVariants. For gnomAD data, only variants located in xGen-captured protein-coding regions were used; filtering was based on GATK metrics obtained from gnomAD and only “PASS” variants were included. SNVs with VQSR <− 20 and indels with VQSR <− 5 were excluded. Variants used for downstream analyses were restricted to the subset called by both *GLnexus* and DeepVariants.

De novo variants were defined as a variant present in the offspring with homozygous reference genotypes in both parents. We used a series of filters to identify de novo variants: VQSR tranche ≤ 99.7 for SNVs and ≤ 99.0 for indels; GATK Fisher Strand ≤ 25; quality by depth ≥ 2. We required the candidate de novo variants in probands to have ≥ 5 reads supporting the alternative allele, ≥ 20% alternative allele fraction, Phred-scaled genotype likelihood ≥ 60 (GQ), and population AF ≤ 0.01% in ExAC and required both parents to have ≥ 10 reference reads, < 5% alternative allele fraction, and GQ ≥ 30.

We used Ensembl Variant Effect Predictor (VEP; Ensemble 93) [41] to annotate variant function and ANNOVAR [42] to aggregate variant population frequencies and in silico predictions of deleteriousness. Rare synonymous variants were further evaluated with SpliceAI [43] to identify cryptic splice site variants (score ≥ 0.5). Rare variants were defined as AF ≤ 0.01% in gnomAD exome_ALL (all ancestries). A total of 18,939 protein-coding genes were identified containing ≥ 1 rare variant, excluding mucin and major histocompatibility complex genes due to low sequence complexity. Deleterious variants were defined as likely gene-disrupting (LGD, including premature stop-gain, frameshift indels, canonical splicing variants, cryptic splice site variants, and exon deletions) or predicted damaging missense (D-Mis) based on gene-specific REVEL score thresholds [18, 44] (see below). All rare inherited and de novo variants in candidate genes were manually inspected using Integrative Genome Viewer (IGV) [45]. Indels were confirmed independently by Sanger sequencing.

### Statistical analysis

To identify novel risk genes for IPAH, we performed a rare variant association test in unrelated participants of European ancestry. Genetic ancestry and relatedness of cases and SPARK controls were checked using Peddy [46], and only unrelated cases (*n* = 2789) and controls (18,819: 11,101 SPARK parents and 7718 gnomAD individuals) were included in the association test. The gnomAD controls were confined to non-Finnish Europeans (NFE). We performed a gene-based case-control test comparing the frequency of rare deleterious variants in PAH cases with unaffected controls. To reduce batch effects in combined datasets from different sources [47], we limited the analysis to regions targeted by xGen and with at least 10× coverage in 90% of samples. We then tested for similarity of the rare synonymous variant rate among cases and controls, assuming that most rare synonymous variants do not have discernible effects on disease risk.

To identify PAH risk genes, we tested the burden of rare deleterious variants (AF ≤ 0.01%, LGD or D-Mis) in each protein-coding gene in cases compared to controls using a variable threshold test [48]. Specifically, we used REVEL [44] scores to predict the deleteriousness of missense variants, searched for a gene-specific optimal REVEL score threshold that maximized the burden of rare deleterious variants in cases compared to controls, and then used permutations to calculate statistical significance as described previously [7] to control the type I error rate. We checked for inflation using a quantile-quantile (Q-Q) plot and calculated the genomic control factor, lambda, using QQperm (https://cran.r-project.org/web/packages/QQperm/QQperm.pdf). Lambda equal to 1 indicates no deviation from the expected distribution. We performed two association tests, one with LGD and D-Mis variants combined and the other with D-Mis variants alone. We defined the threshold for genome-wide significance by Bonferroni correction for multiple testing (*n* = 40,000, 18,939 protein-coding genes containing rare variants times two tests for each gene, yielding a threshold *p* value = 1.25e−6). We used the Benjamini-Hochberg procedure to estimate false discovery rate (FDR) by p.adjust in R.

To test whether recurrent variants in individual genes represented independent mutational events or were due to founder events, we first tested for relatedness among samples using KING [49], in addition to *Peddy* [46]. None of the cases with recurrent variants had any evidence of relatedness. Second, we assessed shared haplotypes of recurrent variant carriers using SHAPEIT2 [50] and the HapMap genetic map [51]. Since all of the recurrent variant carriers were of European ancestry, we restricted the HapMap data to the European population.

To estimate the burden of de novo variants in cases, we calculated the background mutation rate using a previously published tri-nucleotide change table [52, 53] and calculated the rate in protein-coding regions that are uniquely mappable. We assumed that the number of de novo variants of various types (e.g., synonymous, missense, LGD) expected by chance in gene sets or all genes followed a Poisson distribution [52]. For a given type of de novo variant in a gene set, we set the observed number of cases to *m1*, the expected number to *m0*, estimated the enrichment rate by (*m1*/*m0*), and tested for significance using an exact Poisson test (poisson.test in R) with *m0* as the expectation.

### Protein modeling

Homology structures of conserved protein domains in FBLN2 and PDGFD were built using EasyModeller 4.0 [54]. Template structures were downloaded from the protein database (PDB) for endothelial growth factor (EGF, PDB ID 5UK5) and CUB (PDB ID 3KQ4) domains. The template structure for platelet-derived growth factor (PDGF)/vascular EGF (VEGF) was downloaded directly from PrePPI [55, 56].

### Gene expression

Single-cell RNA-seq data of aorta, lung, and heart tissues were obtained from *Tabula Muris*, a transcriptome compendium containing RNA-seq data from ~ 100,000 single cells from 20 adult-staged mouse organs [57]. We chose 14 tissue/cell types including endothelial, cardiac muscle, and stromal cells from the three tissues, restricting the analysis to tissues/cell types for which there was RNA-seq data from at least 70 individual cells (Additional file 1, Supplementary Figure 1). Relative gene expression was based on the fraction of cells with > 0 reads in each cell type. PCA of cell type-specific gene expression profiles was performed using a script available through GitHub [58].

## Results

### Cohort characteristics

Demographic data and mean hemodynamic parameters of the combined US/UK cohort are shown in Table 1. The cohort includes 4241 cases: 54.6% IPAH, 34.8% APAH, 5.9% FPAH, and 4.6% other PAH. Most of the APAH and other PAH cases came from the PAH Biobank and have been described previously [7]. The majority of cases were adult-onset (92.6%) with a mean age-of-diagnosis (by right heart catheterization) of 45.9 ± 20 years (mean ± SD). As expected for adult-onset PAH cohorts [7, 8, 59], the majority of cases were female (75.1%). The genetically determined ancestries were European (74.5%), Hispanic (8.6%), African (8.7%), East Asian (2.5%), and South Asian (2.8%). Hemodynamic data were collected at the time of PAH diagnosis. Diagnostic criteria for PAH is mean pulmonary arterial pressure (mPAP) > 20–25 mmHg [32]. The mPAP and mean pulmonary capillary wedge pressure (mPCWP) for the overall cohort were 51 ± 14 mmHg (mean ± SD) and 10 ± 4 mmHg, respectively, compared to 58 ± 14 mmHg and 10 ± 4 mmHg for FPAH.

**Table 1:** Demographic data and mean hemodynamic parameters from the US/UK PAH cohort*

	All	IPAH	APAH**	FPAH	Other***
**Total,** ***n*** **(%)**	4241	2319 (54.6)	1479 (34.8)	252 (5.9)	191 (4.6)
**Sex,** ***n*** **(%)**					
F	3187 (75.1)	1721 (74.2)	1156 (78.2)	173 (68.6)	137 (71.7)
M	1054 (24.9)	598 (25.8)	323 (21.8)	79 (31.3)	54 (28.3)
F:M ratio	3:1	2.9:1	3.6:1	2.3:1	2.5:1
**Age-of-onset,** ***n*** **(%)**					
Adult (≥ 18 years)	3780 (89.2)	2126 (91.7)	1256 (85.2)	213 (84.5)	185 (96.9)
Child (< 18 years)	457 (10.8)	193 (8.3)	219 (14.8)	39 (15.4)	6 (3.1)
Mean ± SD	45.9 ± 20.0	47.0 ± 19.5	45.2 ± 21.3	36.8 ± 16.8	47.7 ± 15.0
**Ancestry,** ***n*** **(%)**					
EUR	3108 (74.5)	1798 (77.5)	988 (67.0)	166 (86.9)	156 (81.7)
HISP	359 (8.6)	145 (6.3)	186 (12.6)	13 (6.8)	15 (7.9)
AFR	365 (8.7)	166 (7.2)	189 (12.8)	2 (1.0)	8 (4.2)
EAS	104 (2.5)	42 (1.8)	57 (3.9)	1 (0.5)	4 (2.1)
SAS	116 (2.8)	82 (3.5)	28 (1.9)	4 (2.1)	2 (1.0)
Other/unknown	123 (2.9)	85 (3.7)	27 (1.8)	5 (2.6)	6 (3.1)
**Hemodynamic parameters, mean ± SD (*n*)**					
MPAP, mmHg	51 ± 14 (3594)	53 ± 17 (2045)	48 ± 14 (1235)	58 ± 14 (158)	51 ± 12 (156)
*BMPR2*+	59 ± 12 (320)	60 ± 12 (197)	56 ± 16 (11)	58 ± 12 (109)	53 ± 5 (3)
*BMPR2*−	50 ± 14 (3394)	52 ± 14 (1928)	48 ± 14 (1257)	57 ± 17 (65)	51 ± 13 (144)
MPCWP, mmHg	10 ± 4 (3407)	10 ± 24 (1912)	10 ± 4 (1197)	10 ± 4 (151)	11 ± 4 (147)
*BMPR2*+	10 ± 4 ()	10 ± 4 ()	10 ± 3 ()	10 ± 4 ()	8 ± 4 ()
*BMPR2*−	10 ± 4 ()	10 ± 4 ()	10 ± 4 ()	10 ± 4 ()	11 ± 4 ()

*US/UK PAH cohort: 2572 PAH Biobank; 1134 UK NIHR BioResource; 534 CUIMC

**APAH: PAH associated with connective tissue diseases, congenital heart disease, HHT, HIV

***Other: diet- and toxin-induced PAH, non-familial pulmonary veno-occlusive disease/pulmonary capillary hemangiomatosis and one case of persistent pulmonary hypertension of the newborn

Abbreviations: *AFR*, African; *EAS*, East Asian; *EUR*, European; *HISP*, Hispanic; *SAS*, Southeast Asian; *MPAP*, mean pulmonary artery pressure; *MPCWP*, mean pulmonary capillary wedge pressure

A comparison of the clinical characteristics and hemodynamic data for pediatric- versus adult-onset PAH cases is shown in Additional file 2 (Supplementary Table 1). Notably, the female:male ratio among pediatric-onset cases was significantly lower (1.65:1) compared to adult-onset cases (4:1, *p* < 0.0001 by Fisher’s exact test), and children had higher mPAP and mPCWP, decreased cardiac output and increased pulmonary vascular resistance compared to adults at diagnosis (all differences *p* < 0.0001 by Student’s *t* test).

Rare deleterious variants in *BMPR2* were identified in 7.7% of cases overall (209/2318, 9% of IPAH; 108/191, 56.6% of FPAH; and 13/1475, 0.88% of APAH). The variants include LGD and D-Mis variants as well as intragenic or whole gene deletions as previously described [7, 8, 17, 18]. The percentage of *BMPR2* carriers in the US/UK international cohort is lower than previous reports [8, 12] due to the enrichment of APAH cases, rarely caused by *BMPR2* variants [7, 18].

### Identification of novel risk genes: *FBLN2* and *PDGFD*

To perform a combined analysis of US and UK sequencing data, we reprocessed the UK data using our inhouse pipeline, including predictions of missense variant deleteriousness [7]. Quality control procedures included detection of cryptic relatedness among all PAH participants. We performed a gene-based case-control association analysis to identify novel PAH risk genes using only unrelated cases. To control for population stratification, we confined the association analysis to individuals of European ancestry (2789 cases, 18,819 controls) and then screened the whole cohort, including nonEuropeans, for rare deleterious variants in associated genes. As a quality control check for the filtering parameters employed, we compared the frequencies of rare synonymous variants, a variant class that is mostly neutral with respect to disease status, in European cases vs controls. We observed similar frequencies of synonymous variants in cases vs controls (enrichment rate = 1.0, *p* value = 0.28) (Additional file 2, Supplementary Table 2). Furthermore, a gene-level burden test revealed no enrichment of rare synonymous variants in cases (Additional file 1, Supplementary Figure 2). We then proceeded to test for gene-specific enrichment of rare deleterious variants (AF < 0.01%, LGD and D-Mis, or D-Mis only) in cases compared to controls. We note that to improve power, we empirically determined the optimal REVEL score threshold to define deleterious missense variants in a gene-specific manner using a variable threshold test [7]. To account for potential different modes of action for different risk genes, we tested the association twice for each gene: one with LGD and D-Mis variants and the other with D-Mis variants alone. In this approach, LGD and D-Mis together is optimized for complete or partial loss of function; D-Mis alone is optimized for gain of function or dominant negative variants. We set the total number of tests at twice the number of protein-coding genes for multiple test adjustment, a conservative approach considering that the data used in these two tests per gene are not independent. The Q-Q plot of *p* values from tests in all genes shows negligible genomic inflation (Additional file 1, Supplementary Figure 3). Rare deleterious variants in eleven genes were significantly associated (false discovery rate, FDR < 0.1) with PAH. Among these, seven are known or previously reported candidate PAH risk genes: *BMPR2*, *TBX4*, *GDF2*, *ACVRL1*, *SOX17*, *AQP1*, *ATP13A3*, and *KDR*. Three are new candidate genes: *COL6A5* (collagen type VI alpha 5 chain), *JPT2* (Jupiter microtubule-associated homolog 2), and *FBLN2* (fibulin 2).

**Fig. 1:**
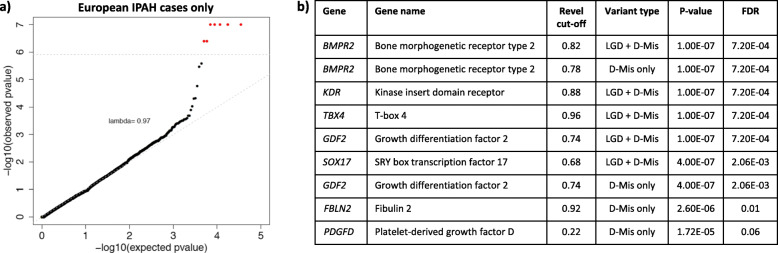
Gene-based association analysis using 1647 European IPAH cases and 18,819 European controls. **a** Results of a binomial test confined to rare, likely gene damaging (LGD) and predicted deleterious missense (D-Mis) variants or D-Mis only variants in 20,000 protein-coding genes. The control group included 11,101 unaffected SPARK parents and 7718 NFE gnomAD v2.1.1 individuals. Horizontal gray line indicates the Bonferroni-corrected threshold for significance. **b** Complete list of top association genes (FDR < 0.1)

The increased power inherent to the combined cohort over the PAH Biobank or UK NIHR BioResource alone is due to a twofold increase in the number of IPAH cases, including the number of European cases used for association analysis. Power analyses indicated that the study had ample power to detect risk genes with large effect size and modest variant allele frequency, or large variant allele frequency and modest effect size, relative to IPAH risk genes identified in smaller cohorts (Additional file 1, Supplementary Figure 4). To take advantage of the increased number of European IPAH cases in the combined cohort, we then restricted the analysis to IPAH. Again, testing for association across all protein-coding genes for 1647 IPAH cases compared to 18,819 controls was generally consistent with expectation under the null model (Fig. 1). Rare predicted deleterious variants in seven genes were significantly associated (FDR < 0.1) with IPAH, including three known genes (*BMPR2*, *GDF2*, and *TBX4*), two recently identified candidate genes (*SOX17* and *KDR*), and two new candidate genes (*FBLN2*, and *PDGFD*, platelet-derived growth factor D). More than 95% of samples for both cases and controls had at least 10× depth of sequence coverage across the target regions for *FBLN2* and *PDGFD* (Additional file 1, Supplementary Figure 5), excluding the possibility that the associations were driven by coverage differences between cases and controls. We also tested for gene-level associations restricting the analysis to European APAH cases (*n* = 998). The Q-Q plot of *p* values from all gene tests is shown in Additional file 1, Supplementary Figure 6. Known PAH gene *ACVRL1* showed association with APAH, consistent with its role in APAH-HHT, but no genes were significantly associated at FDR < 0.1.

*KDR* has recently been implicated as a causal gene for PAH based on a small familial study [60] and our population-based phenotype-driven (SKAT-O) analysis of the UK NIHR BioResource cohort with replication in the PAH Biobank [61]. Both of those analyses were based on protein-truncating variants. Herein, we provide additional statistical evidence based on a burden test including both LGD and D-Mis variants using our variable threshold method. Six cases (5 IPAH, 1APAH-CHD) carry D-Mis variants with empirically determined REVEL > 0.86; details of the variants are provided in Supplementary Table 3. All of the variants are located in the conserved tyrosine kinase domain of the encoded protein (www.uniprot.org). One of the variants, c.3439C>T is recurrent in three cases. There was no evidence of relatedness for these cases, and the relatively short shared haplotype length and common population frequency (Additional file 2, Supplementary Table 4) indicate that the variant occurrences represent independent mutational events rather than being derived from a founder event. None of these cases have variants in other known PAH risk genes. The age-of-onset for the six cases is 57 ± 20 years (mean ± SD, range 25-75 years) and all are of European ancestry. Statistically significant association following Bonferroni correction for multiple testing provides confirmation of the association of *KDR* with PAH using an alternative burden-based statistical method.

**Fig. 2:**
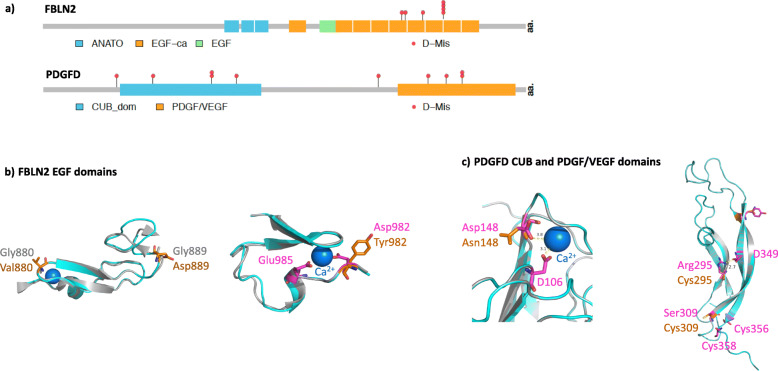
Locations of PAH-associated rare variants within FBLN2 and PDGFD protein structures. **a** Variants and conserved domains within two-dimensional protein structures. The numbers of variants at each amino acid position is indicated along the *y*-axes. D-MIS, predicted deleterious missense; LGD, likely gene-disrupting (stopgain, frameshift, splicing). FBLN2: ANATO, anaphylatoxin-like 2; EGF-ca, calcium-binding endothelial growth factor-like 1; EGF, non-calcium-binding EGF domain. PDGFD: CUB, complement subcomponent; PDGF/VEGF, platelet-derived growth factor/vascular endothelial-derived growth factor domain. **b** FBLN2 residues 858-900: p.(Gly880Val) and p.(Gly889Asp) change the conserved i+2 glycine residues of type II reverse turns within an EGF domain. Residues 981-1011: recurrent p.(Asp982Tyr) changes a residue within the highly conserved DXXE motif/calcium-binding site within an EGF domain. **c** PDGFD residues 43-180: p.(Asp148Asn) predicted to destroy the Ca++ binding site of the CUB domain. Residues 264-364: p.(Arg295Cys) disrupts a hydrogen bond and p.(Ser309Cys) may create a new disulfide bond in the PDGF/VEGF domain

The associations of *FBLN2* and *PGDFD* were both driven by D-Mis variants. We next screened the entire combined cohort, including participants of non-European ancestry, for rare deleterious missense variants in *FBLN2* and *PDGFD*. In total, seven cases carry *FBLN2* variants (6 IPAH, 1 APAH) and ten cases carry *PDGFD* variants (9 IPAH, 1 PAH associated with diet and toxins) (Table 2). Most of the carriers are of European ancestry; one *FBLN2* carrier is of East Asian ancestry and one *PDGFD* carrier is of African ancestry. One *FBLN2* variant ((c.2944G>T; p.(Asp982Tyr)) and two *PDGFD* variants ((c.385G>A; p.(Glu129Lys) and c.961 T>A; p.(Tyr321Asn)) were recurrent in the cohort. Again, there was no evidence of relatedness among these cases, and the shared haplotype characteristics (Additional file 2, Supplementary Table 4) indicate that the variants occurred as independent mutational events. Locations of the predicted damaging missense amino acid residues are shown in Fig. 2. FBLN2 contains multiple endothelial growth factor (EGF) domains, and PDGFD contains a conserved CUB domain and a platelet-derived growth factor (PDGF)/vascular EGF (VEGF) domain. All of the *FBLN2* and eight out of ten *PDGFD* D-Mis variants, occur in conserved protein domains. FBLN2 p.(Gly880Val) and p.(Gly889Asp) replace conserved reverse turn residues in an EGF domain which may change the conformation of the domain and impact protein function (Fig. 2b). Recurrent FBLN2 p.(Asp982Tyr) disrupts a Ca^++^ binding site [62] in another EGF domain (Fig. 2b), which may reduce the affinity and frequency of Ca^++^ binding. PDGFD p.(Asp148Asn) disrupts a Ca^++^ binding site within the CUB domain [63] (Fig. 2c) and recurrent PDFGD p.(Tyr321Asn) is predicted to disrupt a hydrogen bond within the PDGF/VEGF domain (Fig. 2c). In addition, PDGFD p.(Arg295Cys) is located in close proximity to Cys356 and Cys358, potentially introducing new disulfide bonds within the PDGF/VEGF domain.

**Table 2:** Rare predicted deleterious *FBLN2* and *PDGFD* variants* among 4175 PAH cases**

Case ID	Sex	Age_**dx**_	PAH subclass	Ancestry	Gene ***	Exon	Nucleotide change	Amino acid change	Variant type	MAF (gnomAD exomes)	CADD score	Revel
08-018	F	70	IPAH	EUR	*FBLN2*	12	c.2639G>T	p.(Gly880Val)	D-Mis	1.63E−05	27.1	0.94
17-035	F	41	APAH	EAS	*FBLN2*	12	c.2666G>A	p.(Gly889Asp)	D-Mis	–	27.6	0.94
12-207	F	44	IPAH	EUR	*FBLN2*	13	c.2794T>C	p.(Phe923Leu)	D-Mis	2.11E−05	29.6	0.92
23-001	M	66	IPAH	EUR	*FBLN2*	14	c.2944G>T	p.(Asp982Tyr)	D-Mis	1.88E−05	34.0	0.95
29-031	F	57	IPAH	EUR	*FBLN2*	14	c.2944G>T	p.(Asp982Tyr)	D-Mis	1.88E−05	34.0	0.95
34-005	M	69	IPAH	EUR	*FBLN2*	14	c.2944G>T	p.(Asp982Tyr)	D-Mis	1.88E−05	34.0	0.95
W000210	F	52	IPAH	EUR	*FBLN2*	14	c.2944G>T	p.(Asp982Tyr)	D-Mis	1.88E−05	34.0	0.95
W000073	F	40	IPAH	AFR	*PDGFD*	2	c.166G>A	p.(Gly56Ser)	D-Mis	1.99E−05	22.9	0.64
JM950	F	2	IPAH	EUR	*PDGFD*	2	c.250C>T	p.(Arg84Trp)	D-Mis	1.59E−05	16.4	0.51
E012465	F	55	IPAH	EUR	*PDGFD*	3	c.385G>A	p.(Glu129Lys)	D-Mis	–	25.2	0.262
E014342	F	40	IPAH	EUR	*PDGFD*	3	c.385G>A	p.(Glu129Lys)	D-Mis	–	25.2	0.262
E014400	F	43	IPAH	EUR	*PDGFD*	3	c.442G>A	p.(Asp148Asn)	D-Mis	7.97E−06	25.2	0.41
E000844	F	39	IPAH	EUR	*PDGFD*	6	c.770T>C	p.(Leu257Pro)	D-Mis	4.01E−06	31.0	0.62
13-037	M	43	DTOX	EUR	*PDGFD*	6	c.883C>T	p.(Arg295Cys)	D-Mis	4.00E−06	35.0	0.56
23-025	F	41	IPAH	EUR	*PDGFD*	6	c.926C>G	p.Ser309Cys	D-Mis	–	28.4	0.22
E000820	F	73	IPAH	EUR	*PDGFD*	6	c.961T>A	p.(Tyr321Asn)	D-Mis	1.21E−05	33.0	0.34
E010173	F	74	IPAH	EUR	*PDGFD*	6	c.961T>A	p.(Tyr321Asn)	D-Mis	1.21E−05	33.0	0.34

*Rare, deleterious variants defined as gnomAD_exome_ALL AF ≤ 1.00E−04 and LGD or missense with variable REVEL cut-off (*FBLN2* 0.92 and *PDGFD* 0.22)

** Cases are heterozygous for the indicated variants

***Transcripts: *FBLN2* NM_001998.3 and *PDGFD* NM_033135.4

### Clinical phenotypes of *FBLN2* and *PDGFD* variant carriers

The clinical phenotypes of all *FBLN2* and *PDGFD* variant carriers are provided in Table 3. *FBLN2* variant carriers have a similar female:male ratio (2.5:1) compared to the overall cohort (3.1:1) or IPAH alone (2.9:1). *PDGFD* variant carriers are primarily female (9:1) but the distribution is not significantly different from the overall IPAH cohort (*p* = 0.5, Fisher’s exact test). All of the *FBLN2* and *PDGFD* variant carriers have adult-onset disease, with the exception of one pediatric *PDGFD* variant carrier, with no statistically significant differences in mean age-of-onset (53 ± 11 and 45 ± 20 years, respectively) compared to that of the overall cohort (46 ± 20 years) or IPAH alone (47 ± 20 years), excluding *FBLN2* and *PDGFD* variant carriers. *FBLN2* variant carriers exhibit a trend towards increased mean pulmonary artery pressure (62 ± 17, mmHg) and significantly increased mean pulmonary capillary wedge pressure (13 ± 2 mmHg) compared to the overall cohort (51 ± 14, non-significant and 10 ± 4 mmHg, *p* = 0.015 respectively) or IPAH alone (53 ± 17, non-significant and 10 ± 24 mmHg, *p* = 0.01, respectively). *PDGFD* variant carriers have similar pulmonary pressures compared to the overall cohort or IPAH alone. All of the *FBLN2* and *PDGFD* variant carriers were diagnosed with WHO PAH class II or III disease and have no history of lung transplantation. Most of the *FBLN2* and *PDGFD* variant carriers have comorbidities typical of adult IPAH patients [64, 65], including hypertension, hypothyroidism, other pulmonary diseases, and metabolic diseases. Five out of seven *FBLN2* carriers have a diagnosis of systemic hypertension.

**Table 3:** Clinical phenotypes of *FBLN2* and *PDGFD* variant carriers. Sex ratios and mean ± SD diagnostic age and hemodynamic values have been calculated separately for *FBLN2* and *PDGFD* variant carriers

Case ID	Sex	Age_**dx**_ (years)	PAH subclass	Ancestry	Gene	MPAP (mmHg)	MPCWP (mmHg)	WHO functional class	Lung tx	Other medical conditions
08-018	F	70	IPAH	EUR	*FBLN2*	58	14	III	No	HTN, kidney congenital anomaly, Paget’s disease
17-035	F	41	APAH (MCTD)	EAS	*FBLN2*	43	16	III	No	
12-207*	F	44	IPAH	EUR	*FBLN2*	NA	NA	NA	No	HTN, hypothyroidism
23-001	M	66	IPAH	EUR	*FBLN2*	68	10	III	No	HTN, OLD (smoker)
29-031	F	57	IPAH	EUR	*FBLN2*	84	15	NA	No	HTN, mitral valve disease, hypothyroidism, OA, COPD
34-005*	M	69	IPAH	EUR	*FBLN2*	43	14	NA	No	HTN, CAD
W000210	F	52	IPAH	EUR	*FBLN2*	75	11	II	No	Hyposplenism
**Mean ± SD**	**2.5:1**	**53 ± 11**				**62 ± 17**	**13 ± 2**			
W000073*	F	40	IPAH	AFR	*PDGFD*	73	NA	III	No	PFO, bilateral chronic subdural hematoma, hypothyroidism
JM930	F	2	IPAH	EUR	*PDGFD*	39	NA	NA	No	Bronchopulmonary dysplasia
E012465	F	55	IPAH	EUR	*PDGFD*	52	7	III	No	Hypothyroidism, IBS, major depression
E014342*	F	40	IPAH	EUR	*PDGFD*	57	7	III	No	Emphysema
E014400	F	43	IPAH	EUR	*PDGFD*	57	4	III	No	Obesity, T2DM
E000844	F	39	IPAH	EUR	*PDGFD*	51	9	III	No	GERD, asthma, bicornate uterus
13-037	M	43	DTOX	EUR	*PDGFD*	47	7	II	No	None
23-025*	F	41	IPAH	EUR	*PDGFD*	64	NA	III	No	Hypothyroidism
E000820	F	73	IPAH	EUR	*PDGFD*	48	9	II	No	Fatty liver, hypothyroidism, ductal carcinoma, gallstones, superior vena cava and azygos vein thrombosis related to port-a-cath
E010173	F	74	IPAH	EUR	*PDGFD*	32	9	III	No	MPVD, PVD, obesity, T2DM, HTN, chronic renal impairment, hypothyroidism, OA, hypouricemia, major depression
**Mean ± SD**	9:1**	**50 ± 14**				**53 ± 12**	**11 ± 10**			

*Cases with risk variants in additional PAH risk genes: 12-207 (*ABCC8* and *GGCX*), 34-005 (*GGCX*), W000073 (*TBX4*), E014342 (*BMPR2*), 23-025 (*ENG*)

**NS, Fisher’s exact test

Abbreviations: *CAD*, coronary artery disease; *COPD*, chronic obstructive pulmonary disease; *GERD*, gastrooesophageal reflux disease; *HTN*, systemic hypertension; *IBS*, irritable bowel syndrome; *MCTD*, mixed connective tissue disease; *MPVD*, mixed pulmonary valve disease; *OA*, osteoarthritis; *OLD*, obstructive lung disease; *PFO*, patent foramen ovale; *PVD*, peripheral vascular disease; *T2DM*, type 2 diabetes mellitus

### Gene expression patterns of PAH candidate risk genes

**Fig. 3:**
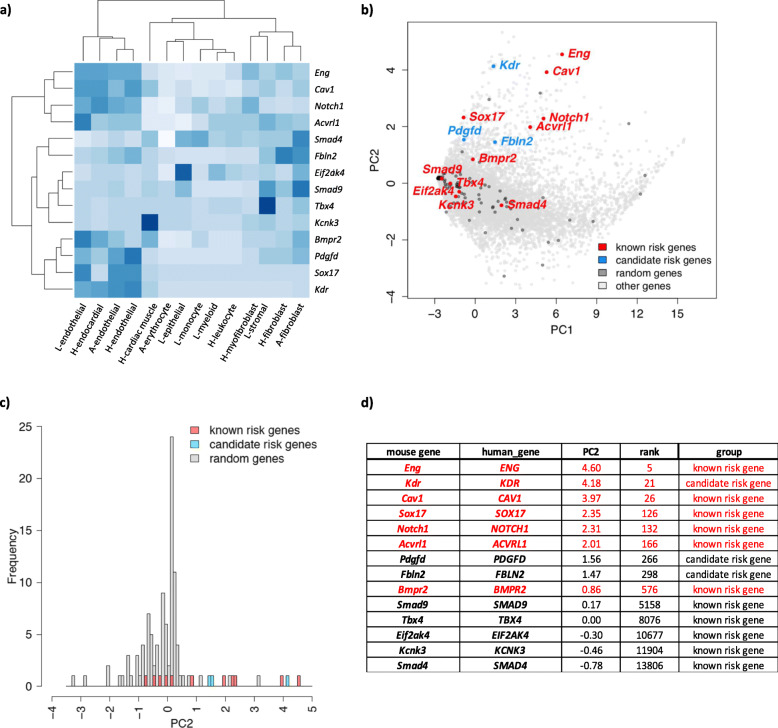
Gene expression patterns of PAH risk genes using murine single-cell RNA-seq data. **a** Heat map showing fraction of cells with > 0 reads in specific cell types of lung, heart, and aorta for 11 known PAH risk genes and 3 new candidate risk genes (*KDR*, *FBLN2*, and *PDGFD*). L, lung; H, heart; A, aorta. **b** PCA analysis of gene expression for PAH risk genes and a set of 100 randomly selected genes, overlaid on a plot of all other 16,744 sequenced genes expressed in both human and mouse cells. **c** Histogram of PC2 values for PAH risk genes and a set of 100 randomly selected genes indicates a right shift for PC2 among PAH risk genes. **d** Relative rank of PC2 values for PAH risk genes among 16,744 sequenced genes expressed in both human and mouse cells.

We hypothesized that PAH risk genes are highly expressed in certain cell types relevant to the disease etiology and that joint analysis of cell type-specific expression data with genetic data could inform cell types associated with disease risk [66]. We obtained single-cell RNA-seq data of aorta, lung, and heart tissues available through the *Tabula Muris* project, a transcriptome compendium containing RNA-seq data from adult-staged mouse organs [57]. We chose 14 tissue/cell types including endothelial, cardiac muscle, and stromal cells as a proxy for the cell types of the pulmonary artery (unavailable). A list of the tissues, cell types, and the number of cells sequenced per tissue/cell type is provided in Additional file 1 (Supplementary Figure 1a). We queried gene expression for twelve known PAH risk genes (*ACVRL1*, *BMPR2*, *CAV1*, *EIF2AK4*, *ENG*, *KCNK3*, *KDR*, *NOTCH1*, *SMAD4*, *SMAD9*, *SOX17*, *TBX4*) and the two new candidate risk genes (*FBLN2*, *PDGFD*). A heat map with hierarchical clustering of relative gene expression is shown in Fig. 3a. The majority of known risk genes (7/12) have relatively high expression in endothelial cells from the three tissues; most others have high expression in tissue-specific cardiac muscle, stromal cells, or fibroblasts. *PDGFD* is located in the same cluster as *BMPR2*, *SOX17*, and *KDR*; these genes are specifically and highly expressed in endothelial cell types. *FBLN2* is highly expressed in both endothelial and fibroblast cell types. We then randomly selected a set of 100 genes without reported associations with PAH and performed PCA of cell type-specific expression profiles of known risk genes and random genes. The second component (PC2) largely separates known risk genes and random genes (Fig. 3b, c). Consistent with hierarchical clustering, endothelial expression in all three tissues was positively correlated with PC2 (Additional file 1, Supplementary Figure 1b). Projecting all protein-coding genes onto PC2, seven of twelve known risk genes are ranked in top 5% among all genes (Fig. 3d) (binomial test: enrichment =20, *p* = 1.6E−05). Two new candidate genes, *FBLN2* and *PDGFD*, are ranked in the top 1.8% of PC2.

### Identification of novel candidate pediatric PAH risk genes by de novo variant analysis

We next focused on pediatric-onset disease, a sub-population in which genetic factors likely play a larger causal role compared to adults. The study was underpowered to carry out a gene-based case-control association analysis due to the relatively small number of pediatric patients (*n* = 442); however, 124 pediatric-onset PAH probands with child-parent trio data were available for de novo variant analysis. The trio cohort consisted mostly of IPAH (55.6%, *n* = 66) and APAH-CHD (37.9%, *n* = 45) cases. We performed a burden test for enrichment of exonic de novo variants among all trio probands by comparing the number of variants observed vs expected based on the background mutation rate. Similar rates of de novo mutations were observed for synonymous, LGD alone, and total missense variants (Table 4). However, there was a significant burden of D-Mis and LGD + D-Mis variants among cases over that expected by chance (Table 4). Inclusion of all protein-coding genes (*n* = 18,939) in the burden test identified 44 rare variants, including 30 D-Mis and 14 LGD, in cases. Confining the test to a set of 5756 genes highly expressed in developing lung (murine E16.5 lung stromal cells) [67] or heart (murine E14.5 heart) [34] revealed a 2.45-fold enrichment of de novo variants among cases (*n* = 19 D-Mis, *n* = 29 LGD + D-Mis) over that expected by chance (*p* = 2.0e−4, *p* = 2.5e−5, respectively). We estimate that 17 of the variants are likely to be implicated in pediatric PAH based upon the enrichment over controls or expected by chance. Among the variants, seven are in known PAH risk genes: four in *TBX4*, two in *BMPR2*, and one in *ACVRL1*. Excluding these known risk genes, there are 22 LGD + D-Mis variants in genes highly expressed in developing heart and lung, still significantly more than expected (enrichment rate = 1.86, *p* = 0.008, 10 expected risk variants). We tested the burden of de novo variants among IPAH cases and observed enrichment of D-Mis and LGD + D-Mis variants similar to that of the overall trio cohort (Additional file 2, Supplementary Table 5). The study was underpowered to detect a significant burden of de novo variants among APAH-CHD cases. The estimated fraction of pediatric IPAH and the overall pediatric cohort explained by de novo variants is 15.2% and 14.5%, respectively. A complete list of all rare, deleterious de novo variants carried by pediatric PAH cases is provided in Additional file 2 (Supplementary Table 6). Similar to other early-onset severe diseases, including CHD and bronchopulmonary dysplasia, the genes identified fit a general pattern for developmental disorders—genes intolerant to loss of function variants (pLI > 0.5 for 40% of the genes) and with known functions as transcription factors, RNA-binding proteins, protein kinases, and chromatin modification. Three of the genes are known CHD risk genes (*NOTCH1*, *PTPN11*, and *RAF1*), and 37% of the genes are known causal genes for a variety of developmental syndromes. Case variant *PTPN11* p.(Asp61Gly) is a known causal variant for Noonan syndrome [68], and *RAF1* p.Pro261 is a hotspot for multiple gain-of-function mutations, including p.(Pro261Thr), causing Noonan syndrome [69].

**Table 4:** Burden of de novo variants in pediatric-onset PAH (*n* = 124 child-parent trios)

	Variant type*	Observed	Expected by chance	Enrichment	***p*** value	Estimated # of risk variants
All genes (18,939 genes)	SYN	42	38.3	1.1	0.51	N/A
	LGD	14	11.8	1.2	0.46	N/A
	MIS	93	84.7	1.1	0.36	N/A
	D-Mis	30	17.3	1.73	0.005	N/A
	LGD + D-Mis	44	28.9	1.52	0.009	15
HLE or HHE** (5756 genes)	SYN	18	14.01	1.28	0.28	N/A
	LGD	10	4.69	2.13	0.03	N/A
	MIS	40	31.68	1.26	0.15	N/A
	D-Mis	19	7.25	**2.62**	**2.0e−4**	**12**
	LGD + D-Mis	29	11.85	**2.45**	**2.5e−5**	**17**

* *SYN*, synonymous; *LGD*, likely gene-disrupting; *MIS*, missense; *D-Mis*, deleterious missense based on REVEL > 0.5

**HLE, high lung expression (murine E16.5 lung stromal cells); *HHE*, high heart expression (murine E14.5 heart)

### Clinical phenotypes of pediatric de novo variant carriers

Among the 36 patients who carry LGD or D-Mis de novo variants (Additional file 2, Supplementary Table 7), there is a 1.8:1 ratio of females to males, a mean age-of-onset of 5.4 ± 4.6 years, 50% of the cases (*n* = 18) have a diagnosis of IPAH, 33.3% (*n* = 12) APAH-CHD and an overlapping but distinct 36.1% of cases have other congenital or growth and development anomalies. *NOTCH1* variant carrier, JM1357, has a diagnosis of APAH-CHD with tetralogy of Fallot, and a recent exome sequencing study of ~ 800 tetralogy of Fallot cases identified *NOTCH1* as the top association signal [70]. *PTPN11* variant carrier, JM155, has a diagnosis of APAH-CHD associated with Noonan syndrome and the c.182A>G variant is known to be pathogenic in Noonan syndrome. Variants in *PSMD12* cause Stankiewicz-Isidor syndrome, sometimes associated with congenital heart defects, and variant carrier 06-095 has a diagnosis of APAH-CHD. Hemodynamic data for the de novo variant carriers (Additional file 2, Supplementary Table 7) was similar to that of all pediatric cases in the cohort (Additional file 2, Supplementary Table 1).

## Discussion

Combined analysis of a large US/UK cohort enriched in adult-onset IPAH cases enabled identification of five known and two new IPAH candidate risk genes with FDR < 0.1: *FBLN2* and *PDGFD* are the new genes. The association was based on a gene-level case-control analysis of 1647 unrelated European IPAH cases. The variants contributing to *FBLN2* and *PDGFD* associations are D-Mis variants predicted to alter highly conserved protein conformation, Ca^++^ binding sites, or intramolecular binding sites within conserved protein domains, likely leading to important structural changes in critical domains. The non-founder nature of recurrent *FBLN2* p.(Asp982Tyr) (*n* = 4 cases), and two *PDGFD* variants recurrent in two unrelated cases each, adds further support for pathogenicity of these alleles. In addition, we confirmed the recent association of *KDR* with PAH [60, 61] based on an alternative statistical approach. We further show that all three of these candidate genes have high expression in lung and heart endothelial cell types, similar to other well established risk genes (*BMPR2* and *SOX17*), further supporting the plausibility of these genes contributing to PAH risk. De novo variant analysis of pediatric-onset PAH (124 trios) showed a 2.45× enrichment of rare deleterious exonic variants, indicating that de novo variants contribute to ~ 15% of pediatric cases across PAH subtypes. The de novo variants implicate new candidate risk genes likely unique to pediatric PAH, but some of the molecular pathways may inform both pediatric- and adult-onset PAH.

*FBLN2* encodes an extracellular matrix protein important for elastic fiber formation and regulation of cell motility, proliferation, and angiogenesis. *FBLN2* is expressed in the lung vasculature but most studies have focused on gene expression in the heart vasculature. In mice, *Fbln2* is expressed in epithelial-mesenchymal transformation during embryonic heart development and is upregulated postnatally throughout coronary vasculogenesis and angiogenesis when transformed mesenchymal cells migrate to the extracellular matrix [71, 72]. *Fbln2*^*-/-*^ mice are viable, fertile, and have intact elastic fiber formation, attributable to compensation by the more widely expressed *Fbln1* gene [73, 74]. However, *Fbln2* expression is required for angiotensin II-induced TGFβ signaling and cardiac fibrosis [75]. In humans, *FBLN2* variants have been associated with atrioventricular septal defects [76] and intracranial aneurysm [77], providing additional support for a role in vascular remodeling. We hypothesize that, in the pulmonary vasculature, gain of function variants may lead to increased TGF-β signaling, increased proliferation and medial hypertrophy. The FBLN2 protein contains 10 consecutive EGF protein-protein interaction domains, nine of which are calcium-binding. All seven of the case variants are missense variants, two of which are predicted to alter the conformation of an EGF domain, and a recurrent variant carried by four cases is predicted to disrupt the Ca^++^ binding site of another EGF domain. The carriers of *FBLN2* variants have adult-onset disease with mean age-of-onset similar to the overall cohort or IPAH alone. Five of seven carriers also have a diagnosis of systemic hypertension (HTN), and it is possible that gene damaging variants in *FBLN2* contribute to the development of HTN. However, given the frequency of HTN in the overall US/UK combined cohort (32% for adult-onset IPAH; similar to that reported in the REVEAL registry [65]), there may be other age-related genetic and environmental factors contributing to HTN. Finally, two of our cohort cases, 08-018 and 29-031, have additional diagnoses of renal or heart anomalies, and *FLBN2* has been identified as a key regulator of development in those tissues [78–80].

*PDGFD* is a member of the *PDGF* family that functions in recruiting cells of mesenchymal origin during development or to sites of injury [81]. *PDGFD* is widely expressed including arterial endothelial cells, adventitial pericytes and smooth muscle cells, lung endothelial cells, and smooth muscle cell progenitors of distal pulmonary arterioles. Secreted PDGFD specifically binds PDGFRβ, a widely expressed protein that co-localizes with PDGFD in vascular smooth muscle cells. *Pdgfd* knockout mice are phenotypically normal with the exception of a modest increase in systemic blood pressure [82], However, cardiac-specific *PDGFD* transgenic mice, overexpressing the active core domain of human PDGFD in the heart, exhibit vascular smooth muscle cell proliferation, vascular remodeling with wall thickening, severe cardiac fibrosis, heart failure, and premature death [83]. While effects of *Pdgfd* overexpression on the pulmonary vasculature have not been investigated, the cardiac vasculature data are consistent with a gain of function mechanism. Further evidence for the role of PDGFD as an effector molecule in cardiovascular diseases and cancer has been reviewed [81, 84, 85]. Full-length PDGFD contains two conserved protein domains, an autoinhibitory CUB domain and an enzymatic PDGF/VEGF domain; the protein undergoes proteolytic cleavage at Arg247 or Arg249 to produce an active growth factor promoting angiogenesis and vascular muscularization [86]. All ten of the case variants are missense variants; four reside in the CUB domain and five reside in the active processed protein. Variant p.(Asp148Asn), carried by two patients, is predicted to disrupt the Ca^++^ binding site of the CUB domain; variants p.(Arg295Cys) and p.(Ser309Cys), carried by one and two patients respectively, are predicted to alter the conformation of the PDGF/VEGF domain. All but one of the *PDGFD* variant carriers have adult-onset disease with mean age-of-onset similar to the overall cohort or IPAH alone. Four out of ten of the *PDGFD* variant carriers have additional diagnoses of other pulmonary fibrotic and/or vascular fibrotic diseases including bronchopulmonary dysplasia, emphysema, asthma, and one patient (E010173) with both mixed pulmonary valve disease and peripheral vascular disease (Table 3). Targeting the PDGF pathway with small molecule inhibitors of tyrosine kinase is an active area of investigation and several inhibitors are FDA-approved [87]. Notably, imatinib reduced cardiac fibroblast proliferation and PDGFD expression 15-fold [88]; data regarding effects on pulmonary arterial smooth muscle cells are warranted. A limitation of tyrosine kinase inhibitors is that they target multiple tyrosine kinases. Sequestering PDGFD with neutralizing antibodies or DNA/RNA aptamers, or preventing PDGFD-PDGFRβ interaction via oligonucleotides, may provide more specific targeting.

To test the plausibility of the new candidate PAH genes identified by association analysis, we leveraged publicly available single-cell RNA-seq data. *PDGFD*, and recently identified *KDR*, have very similar expression patterns as *BMPR2* and *SOX17*, two established PAH genes. PCA indicated that the PAH risk genes can largely be separated from non-risk genes based on PC2. The majority of known PAH risk genes rank in the top 5% of PC2 among 16,744 genes queried, and the new genes—*FBLN2* and *PDGFD*—rank within the top 1.8%, providing support for their candidacy as PAH risk genes. Other risk genes, like *KCNK3* and *EIF2AK4*, exert important PAH-related functions in cell types other than endothelial cells, and GDF2 is excreted from liver; thus, it will be important to consider expression patterns on a gene-specific basis. In addition, the dataset utilized in this study was based on adult-staged murine cells and is not well-suited for developmental genes such as *TBX4* and other genes likely to contribute to pediatric-onset disease. Thus, additional datasets from different time points are needed.

Rare deleterious variants in established PAH genes are clearly pathogenic based on segregation analyses, enrichment of rare deleterious variants in PAH cases compared to controls with replication over time, and demonstrated loss of function or aberrant function in vitro, in vivo (model organisms), or ex vivo [6]. However, none of the PAH genes are fully dominant and many carriers are never diagnosed with PAH. *BMPR2* variants exhibit variable penetrance with ~ 14% penetrance in male carriers and 42% in females, suggesting sex as an important modifier of penetrance [6]. Other factors influencing penetrance likely include additional genetic factors, epigenetic factors [89], environmental factors, and gene × environment interactions. Explicit testing of oligogenicity for rare diseases, or gene-environment interactions, require much larger cohorts than those currently available for PAH. However, as more putative risk genes are identified and more PAH cases are studied [7, 8, 15, 17], formal tests to assess the contributions of multiple genetic and environmental risks should be included. In the current study, five of the seventeen cases identified with rare deleterious variants in *FBLN2* or *PDGFD* also carry variants in one or two established or recently reported candidate risk genes. For example, participant 12-207 carries variants in *FBLN2* as well as *ABCC8* and *GGCX*, and participant W000073 carries variants in *PDGFD* and *TBX4*. We acknowledge the possibility that at least some of the variants identified to date may not be causal and that the relative contribution of individual variants requires further investigation. How multiple rare variants interact to affect PAH pathogenesis, penetrance, endophenotypes, or clinical outcomes will require much larger cohorts and will be one of the major aims of future large international consortia.

Our pediatric data indicate that children present with slightly higher mean pulmonary arterial pressure, decreased cardiac output, and increased pulmonary vascular resistance compared to adults at diagnosis. The early age-of-onset and increased severity of clinical phenotypes suggest that there may be differences in the genetic underpinnings. De novo mutations have emerged as an important class of genetic factors underlying rare diseases, especially early-onset severe conditions [34, 90], due to strong negative selection decreasing reproductive fitness [91]. Pediatric-onset PAH fits this category of diseases based on the high mortality during childhood [92–96]. Previously, we reported an enrichment of de novo variants in a cohort of 34 PAH probands with trio data [17]. We have now expanded this analysis to 124 trios with pediatric-onset PAH probands and confirmed the 2.45× enrichment of de novo variants in cases compared to the expected rate. Seven of the variant carriers have variants in known PAH risk genes (*TBX4*, *BMPR2*, *ACVRL1*), and three of the APAH-CHD variant carriers have variants in known CHD or CHD-associated risk genes (*NOTCH1*, *PTPN11*, *PSMD12*). We previously reported rare inherited LGD or D-Mis variants in CHD risk genes *NOTCH1* (*n* = 5), *PTPN11* (*n* = 1), and *RAF1* (*n* = 2) carried by APAH-CHD cases [18]. Specific inhibition of the protein encoded by *PTPN11* (SHP2) [97], and induction of mir-204 which negatively targets SHP2 [98], improved right ventricular function in the monocrotaline rat model of PAH, suggesting a more general role of *PTPN11* in PAH.

At least eight of the other genes with case-derived de novo variants have plausible roles in lung/vascular development but have not been previously implicated in PAH: *AMOT* (angiomotin), *CSNK2A2* (casein kinase 2 alpha 2), *HNRNPF* (heterogeneous nuclear ribonucleoprotein F), *HSPA4* (heat shock protein family A member 4), *KDM3B* (lysine demethylase 3B), *KEAP1* (kelch-like ECH-associated protein 1), *MECOM* (MDS1 and EVI1 complex locus), and *ZMYM2* (zinc finger MIM-type containing 2). A common single-nucleotide polymorphism in *MECOM* has been implicated in systemic blood pressure [99]. *KEAP1* encodes the principle negative regulator of transcription factor NF-E2 p45-related factor 2 (NRF2). The NRF2-KEAP1 partnership provides an evolutionarily conserved cytoprotective mechanism against oxidative stress. Under normal conditions, KEAP1 targets NRF2 for ubiquitin-dependent degradation and represses NRF2-dependent gene expression. KEAP1 is ubiquitously expressed and aberrant oxidative stress response in the pulmonary vasculature is a recognized mechanism underlying PAH. Together, our analysis indicates that 15% of PAH cases are attributable to de novo variants. A larger pediatric cohort will be necessary to confirm some of these genes via replication and identify additional new genes and pathways that will likely be unique to children and not identifiable through studies of adults with PAH.

## Conclusions

We have identified *FBLN2* and *PDGFD* as new candidate risk genes for adult-onset IPAH, accounting for 0.26% and 0.35% of 2318 IPAH cases in the US/UK combined cohort, respectively. We note that five of seven *FBLN2* variant carriers also have a diagnosis of systemic hypertension. A few cases carry rare variants in more than one PAH risk gene, consistent with oligogenic nature of PAH in some individuals. Analysis of single-cell RNA-seq data shows that the new candidate genes have similar expression patterns to well-known PAH risk genes, providing orthogonal support for the new genes and providing more mechanistic insight. We estimate that ~ 15% of all pediatric cases are attributable to de novo variants and that many of these genes are likely to have important roles in developmental processes. Larger adult and pediatric cohorts are needed to better clinically characterize these rare genetic subtypes of PAH.

## Supplementary Information

**Additional file 1: Supplementary Figure 1.** Selection of single-cell RNAseq data. **Supplementary Figure 2.** Gene-level burden test for rare synonymous variants. **Supplementary Figure 3.** Gene-based association analysis for all PAH subclasses. **Supplementary Figure 4.** Power analysis. **Supplementary Figure 5.** Depth of coding sequence coverage for *FBLN2* and *PDGFD*. **Supplementary Figure 6.** Gene-based association analysis for APAH alone.

**Additional file 2: Supplementary Table 1.** Clinical characteristics and hemodynamic parameters of child- vs adult-onset PAH cases. **Supplementary Table 2.** Similar frequency of rare synonymous variants among cases and controls. **Supplementary Table 3.** Rare predicted deleterious *KDR* missense variants. **Supplementary Table 4.** Haplotype analysis of PAH cases with recurrent variants in new candidate genes. **Supplementary Table 5.** Burden of de novo variants in pediatric-onset IPAH. **Supplementary Table 6.** Rare de novo risk variants identified in pediatric-onset PAH. **Supplementary Table 7.** Clinical characteristics of pediatric PAH cases with rare de novo variants.

## Data Availability

The datasets used and/or analyzed during the current study are available via contact with the senior authors. For PAH Biobank data, a Confidentiality Agreement with the collaborating Regeneron Sequencing Center grants to Dr. Nichols a nonexclusive, worldwide, irrevocable, perpetual, royalty-free sublicensable license to access and use the genomic data for any and all purposes. Therefore, while the PAH Biobank data are not uploaded to a publicly available database, direct access to the data are granted by the corresponding author on reasonable request who has full administrative access to all of the data. The data from the NIHRBR-RD study have been deposited in the European Genome-Phenome Archive [100]. Data from most of the affected participants in the US/UK combined cohort were included in previous publications from our group [7, 8, 17, 18, 27, 61, 100]. The following scripts are available: association test of rare variants with variable threshold of predicted functional scores [48] and principle component analysis of rare variants from Tabula Muris [58].
