# Correction to: Rare variant analysis of 4241 pulmonary arterial hypertension cases from an international consortium implicates FBLN2, PDGFD, and rare de novo variants in PAH

**DOI:** 10.1186/s13073-021-00915-w

**Published:** 2021-06-22

**Authors:** Na Zhu, Emilia M. Swietlik, Carrie L. Welch, Michael W. Pauciulo, Jacob J. Hagen, Xueya Zhou, Yicheng Guo, Johannes Karten, Divya Pandya, Tobias Tilly, Katie A. Lutz, Jennifer M. Martin, Carmen M. Treacy, Erika B. Rosenzweig, Usha Krishnan, Anna W. Coleman, Claudia Gonzaga-Jauregui, Allan Lawrie, Richard C. Trembath, Martin R. Wilkins, Regeneron Genetics Center, PAH Biobank Enrolling Centers’ Investigators, NIHR BioResource for Translational Research - Rare Diseases, National Cohort Study of Idiopathic and Heritable PAH, Nicholas W. Morrell, Yufeng Shen, Stefan Gräf, William C. Nichols, Wendy K. Chung

**Affiliations:** 1 Department of Pediatrics, Columbia University Irving Medical Center, 1150 St. Nicholas Avenue, Room 620, New York, NY 10032 USA; 2 Department of Systems Biology, Columbia University, New York, NY USA; 3 Department of Medicine, University of Cambridge, Cambridge Biomedical Campus, Cambridge, UK; 4 Division of Human Genetics, Cincinnati Children’s Hospital Medical Center, Cincinnati, OH USA; 5 Department of Pediatrics, University of Cincinnati College of Medicine, Cincinnati, OH USA; 6 42Genetics, Belfast, Ireland; 7 NIHR BioResource for Translational Research, Cambridge Biomedical Campus, Cambridge, UK; 8 Regeneron Pharmaceuticals, New York, NY USA; 9 Department of Infection, Immunity and Cardiovascular Disease, University of Sheffield, Sheffield, UK; 10 Department of Medical and Molecular Genetics, King’s College London, London, UK; 11 National Heart & Lung Institute, Imperial College London, London, UK; 12 www.pahbiobank.org, Cincinnati, OH USA; 13 University of Cambridge and Cambridge University Hospitals NHS Foundation Trust, Cambridge Biomedical Campus, Cambridge, UK; 14 www.ipahcohort.com, Cambridge, UK; 15 Addenbrooke’s Hospital NHS Foundation Trust, Cambridge Biomedical Campus, Cambridge, UK; 16 Royal Papworth Hospital NHS Foundation Trust, Cambridge Biomedical Campus, Cambridge, UK; 17 Department of Biomedical Informatics, Columbia University, New York, NY USA; 18 Department of Haematology, University of Cambridge, Cambridge Biomedical Campus, Cambridge, UK; 19 Herbert Irving Comprehensive Cancer Center, Columbia University Irving Medical Center, New York, NY USA; 20 Department of Medicine, Columbia University Irving Medical Center, New York, NY USA

**Correction to: Genome Med 13, 80 (2021)**

**https://doi.org/10.1186/s13073-021-00891-1**

It was highlighted that the original article [1] contained an error in the name of Claudia Gonzaga-Jauregui. It was incorrectly captured as Gonzaga-Juaregui. The original article has been updated.
