# Supplementary material for: Rare variant analysis of 4241 pulmonary arterial hypertension cases from an international consortium implicates FBLN2, PDGFD, and rare de novo variants in PAH
Source: Genome Med. 2021 May 10;13:80. doi: 10.1186/s13073-021-00891-1 (PMC8112021; doi:10.1186/s13073-021-00891-1)
Supplement: Supplementary file 1 — Additional file 1: Supplementary Figure 1. Selection of single-cell RNAseq data. Supplementary Figure 2. Gene-level burden test for rare synonymous variants. Supplementary Figure 3. Gene-based association analysis for all PAH subclasses. Supplementary Figure 4. Power analysis. Supplementary Figure 5. Depth of coding sequence coverage for FBLN2 and PDGFD. Supplementary Figure 6. Gene-based association analysis for APAH alone. [file 13073_2021_891_MOESM1_ESM.pptx]

## Slide 1
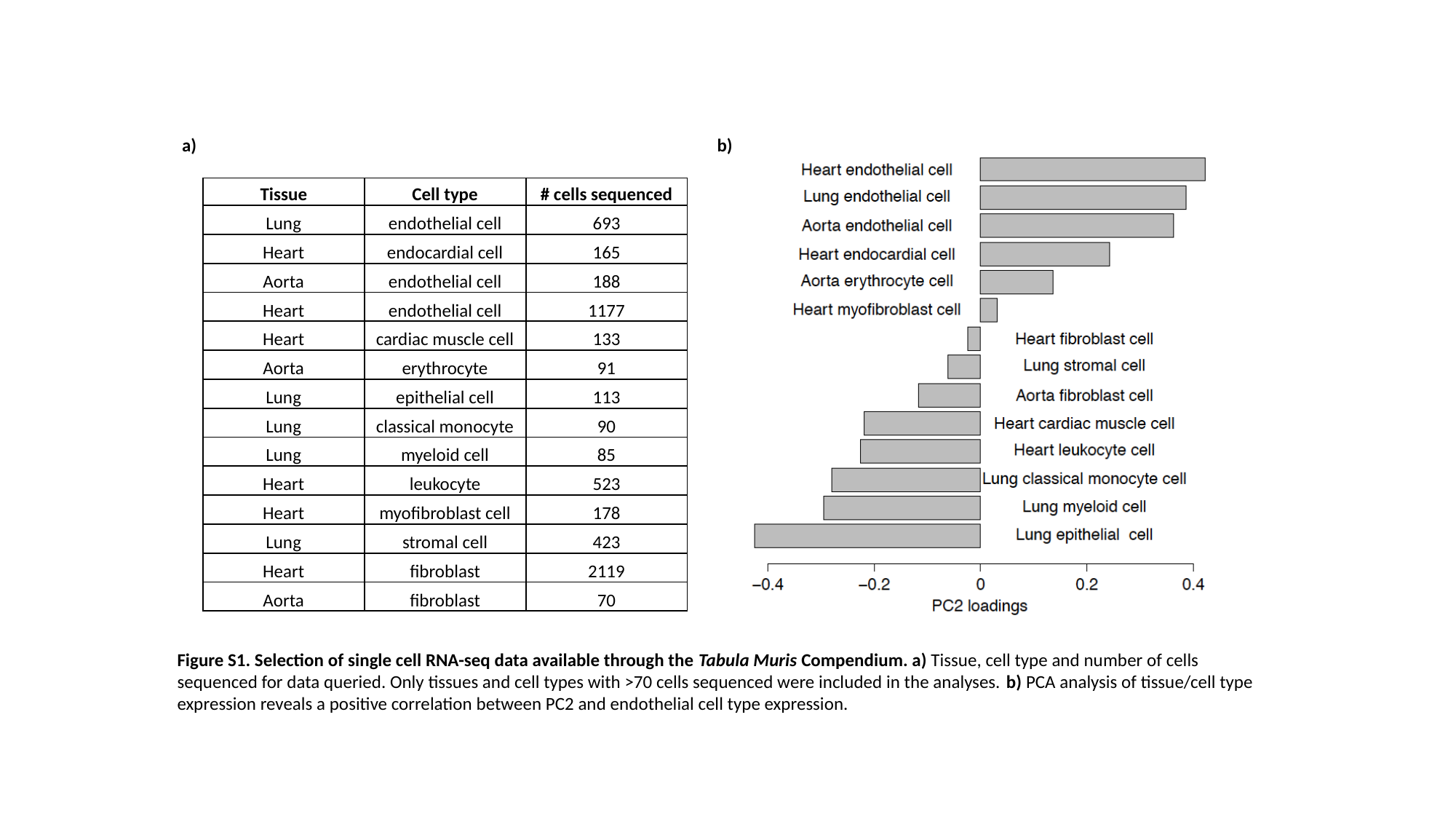

b)
a)
| Tissue | Cell type | # cells sequenced |
| --- | --- | --- |
| Lung | endothelial cell | 693 |
| Heart | endocardial cell | 165 |
| Aorta | endothelial cell | 188 |
| Heart | endothelial cell | 1177 |
| Heart | cardiac muscle cell | 133 |
| Aorta | erythrocyte | 91 |
| Lung | epithelial cell | 113 |
| Lung | classical monocyte | 90 |
| Lung | myeloid cell | 85 |
| Heart | leukocyte | 523 |
| Heart | myofibroblast cell | 178 |
| Lung | stromal cell | 423 |
| Heart | fibroblast | 2119 |
| Aorta | fibroblast | 70 |
Figure S1. Selection of single cell RNA-seq data available through the Tabula Muris Compendium. a) Tissue, cell type and number of cells sequenced for data queried. Only tissues and cell types with >70 cells sequenced were included in the analyses. b) PCA analysis of tissue/cell type expression reveals a positive correlation between PC2 and endothelial cell type expression.

## Slide 2
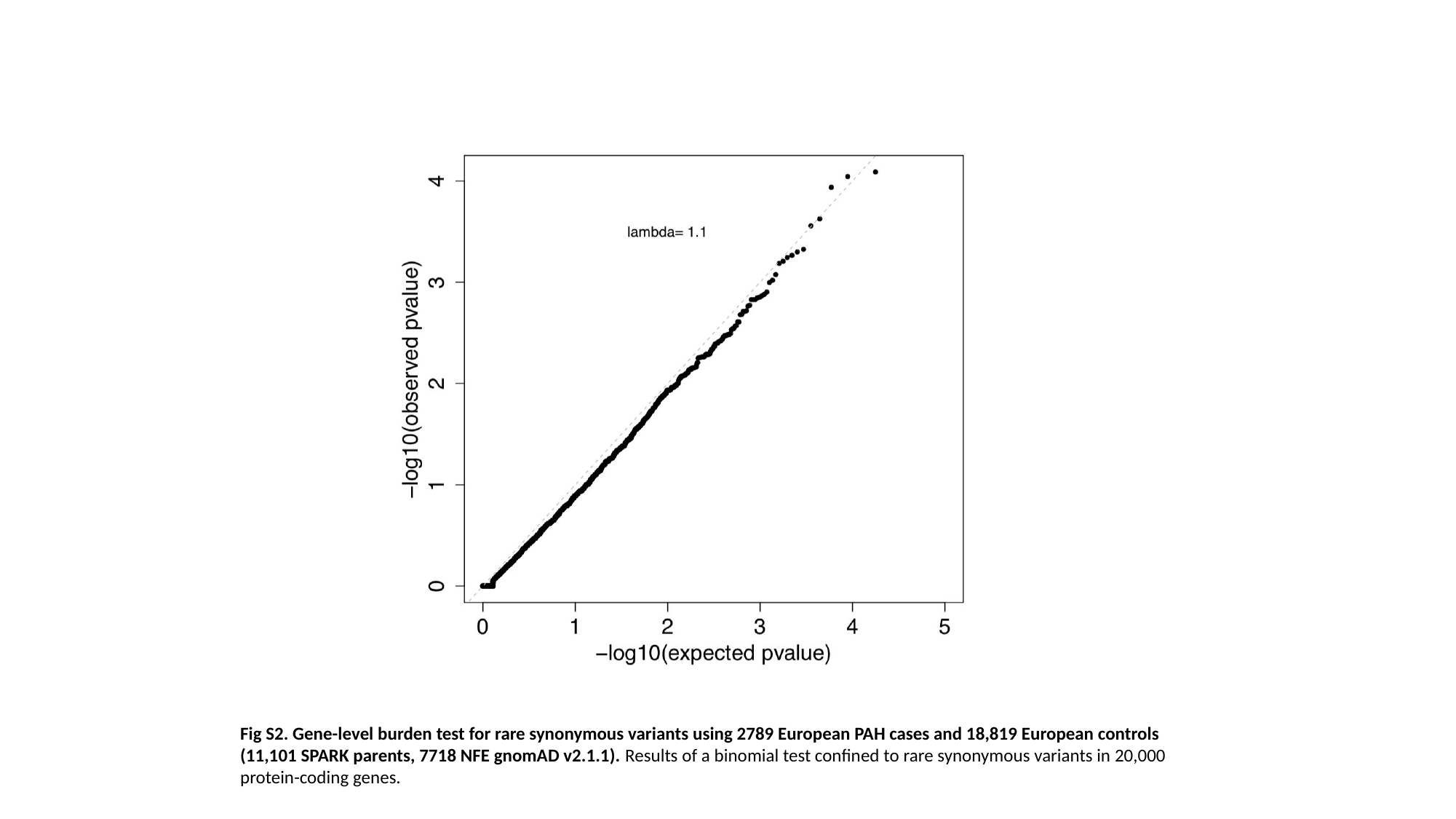

Fig S2. Gene-level burden test for rare synonymous variants using 2789 European PAH cases and 18,819 European controls (11,101 SPARK parents, 7718 NFE gnomAD v2.1.1). Results of a binomial test confined to rare synonymous variants in 20,000 protein-coding genes.

## Slide 3
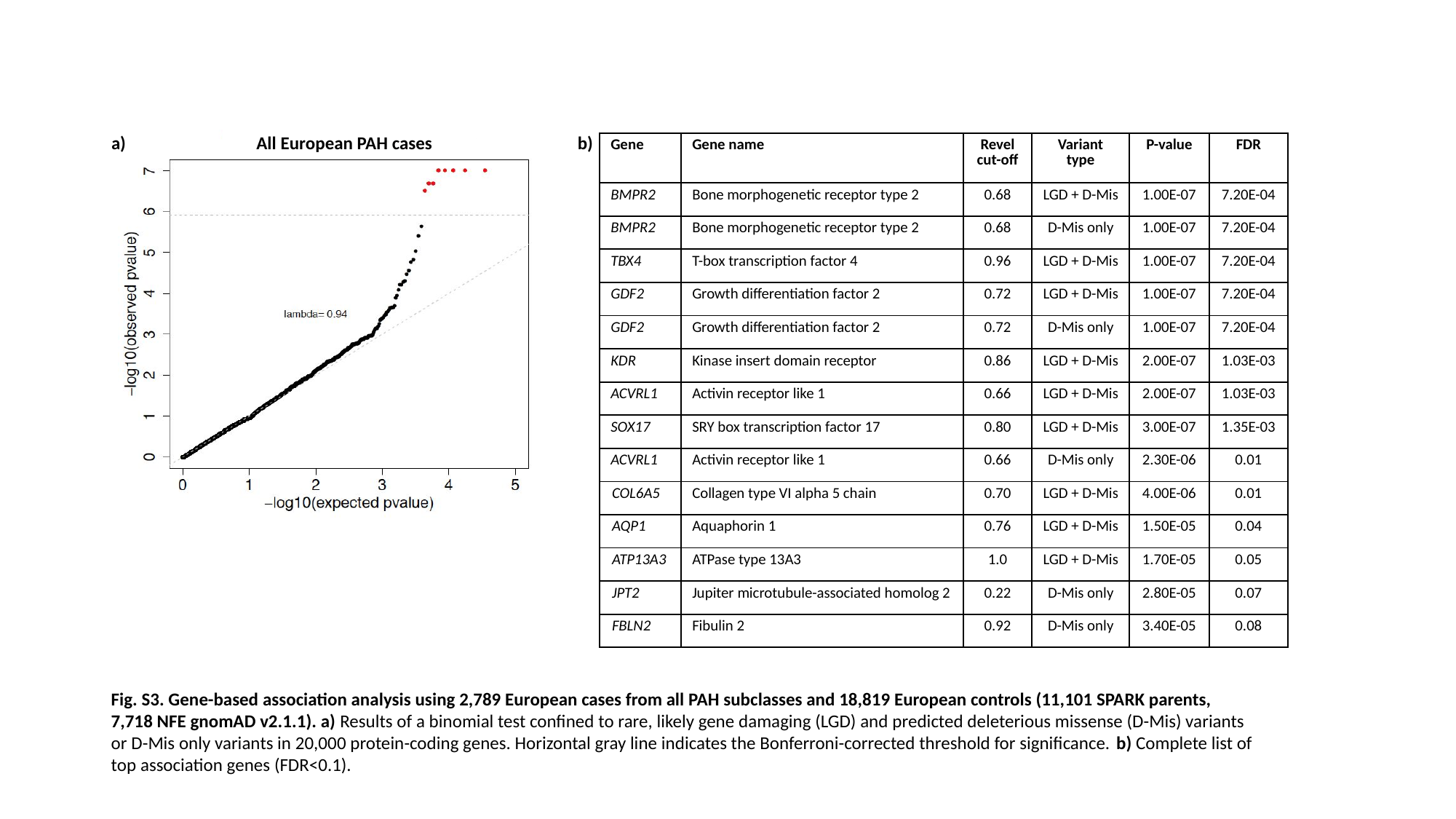

a)
b)
All European PAH cases
| Gene | Gene name | Revel cut-off | Variant type | P-value | FDR |
| --- | --- | --- | --- | --- | --- |
| BMPR2 | Bone morphogenetic receptor type 2 | 0.68 | LGD + D-Mis | 1.00E-07 | 7.20E-04 |
| BMPR2 | Bone morphogenetic receptor type 2 | 0.68 | D-Mis only | 1.00E-07 | 7.20E-04 |
| TBX4 | T-box transcription factor 4 | 0.96 | LGD + D-Mis | 1.00E-07 | 7.20E-04 |
| GDF2 | Growth differentiation factor 2 | 0.72 | LGD + D-Mis | 1.00E-07 | 7.20E-04 |
| GDF2 | Growth differentiation factor 2 | 0.72 | D-Mis only | 1.00E-07 | 7.20E-04 |
| KDR | Kinase insert domain receptor | 0.86 | LGD + D-Mis | 2.00E-07 | 1.03E-03 |
| ACVRL1 | Activin receptor like 1 | 0.66 | LGD + D-Mis | 2.00E-07 | 1.03E-03 |
| SOX17 | SRY box transcription factor 17 | 0.80 | LGD + D-Mis | 3.00E-07 | 1.35E-03 |
| ACVRL1 | Activin receptor like 1 | 0.66 | D-Mis only | 2.30E-06 | 0.01 |
| COL6A5 | Collagen type VI alpha 5 chain | 0.70 | LGD + D-Mis | 4.00E-06 | 0.01 |
| AQP1 | Aquaphorin 1 | 0.76 | LGD + D-Mis | 1.50E-05 | 0.04 |
| ATP13A3 | ATPase type 13A3 | 1.0 | LGD + D-Mis | 1.70E-05 | 0.05 |
| JPT2 | Jupiter microtubule-associated homolog 2 | 0.22 | D-Mis only | 2.80E-05 | 0.07 |
| FBLN2 | Fibulin 2 | 0.92 | D-Mis only | 3.40E-05 | 0.08 |
Fig. S3. Gene-based association analysis using 2,789 European cases from all PAH subclasses and 18,819 European controls (11,101 SPARK parents, 7,718 NFE gnomAD v2.1.1). a) Results of a binomial test confined to rare, likely gene damaging (LGD) and predicted deleterious missense (D-Mis) variants or D-Mis only variants in 20,000 protein-coding genes. Horizontal gray line indicates the Bonferroni-corrected threshold for significance. b) Complete list of top association genes (FDR<0.1).

## Slide 4
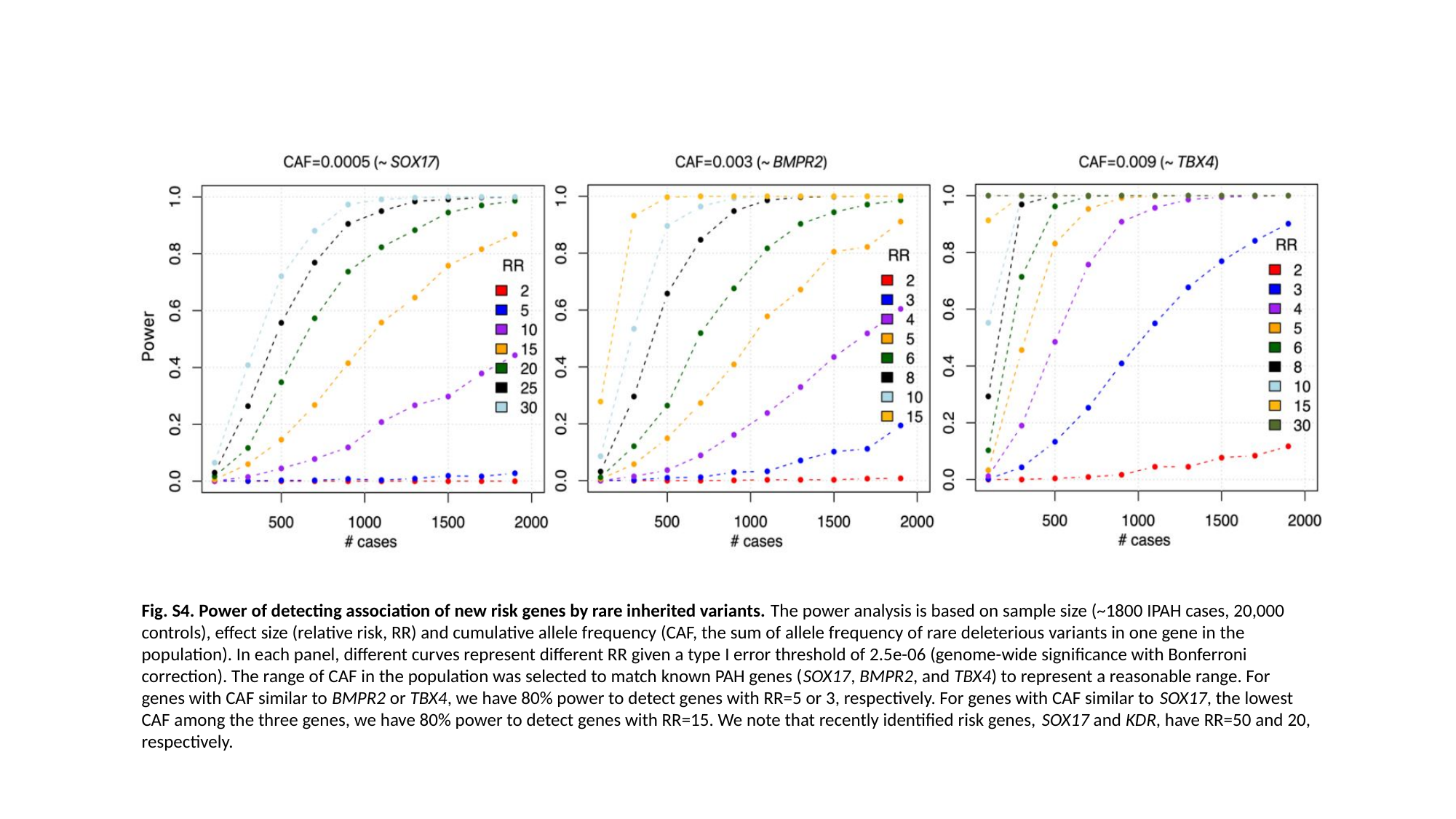

Fig. S4. Power of detecting association of new risk genes by rare inherited variants. The power analysis is based on sample size (~1800 IPAH cases, 20,000 controls), effect size (relative risk, RR) and cumulative allele frequency (CAF, the sum of allele frequency of rare deleterious variants in one gene in the population). In each panel, different curves represent different RR given a type I error threshold of 2.5e-06 (genome-wide significance with Bonferroni correction). The range of CAF in the population was selected to match known PAH genes (SOX17, BMPR2, and TBX4) to represent a reasonable range. For genes with CAF similar to BMPR2 or TBX4, we have 80% power to detect genes with RR=5 or 3, respectively. For genes with CAF similar to SOX17, the lowest CAF among the three genes, we have 80% power to detect genes with RR=15. We note that recently identified risk genes, SOX17 and KDR, have RR=50 and 20, respectively.

## Slide 5
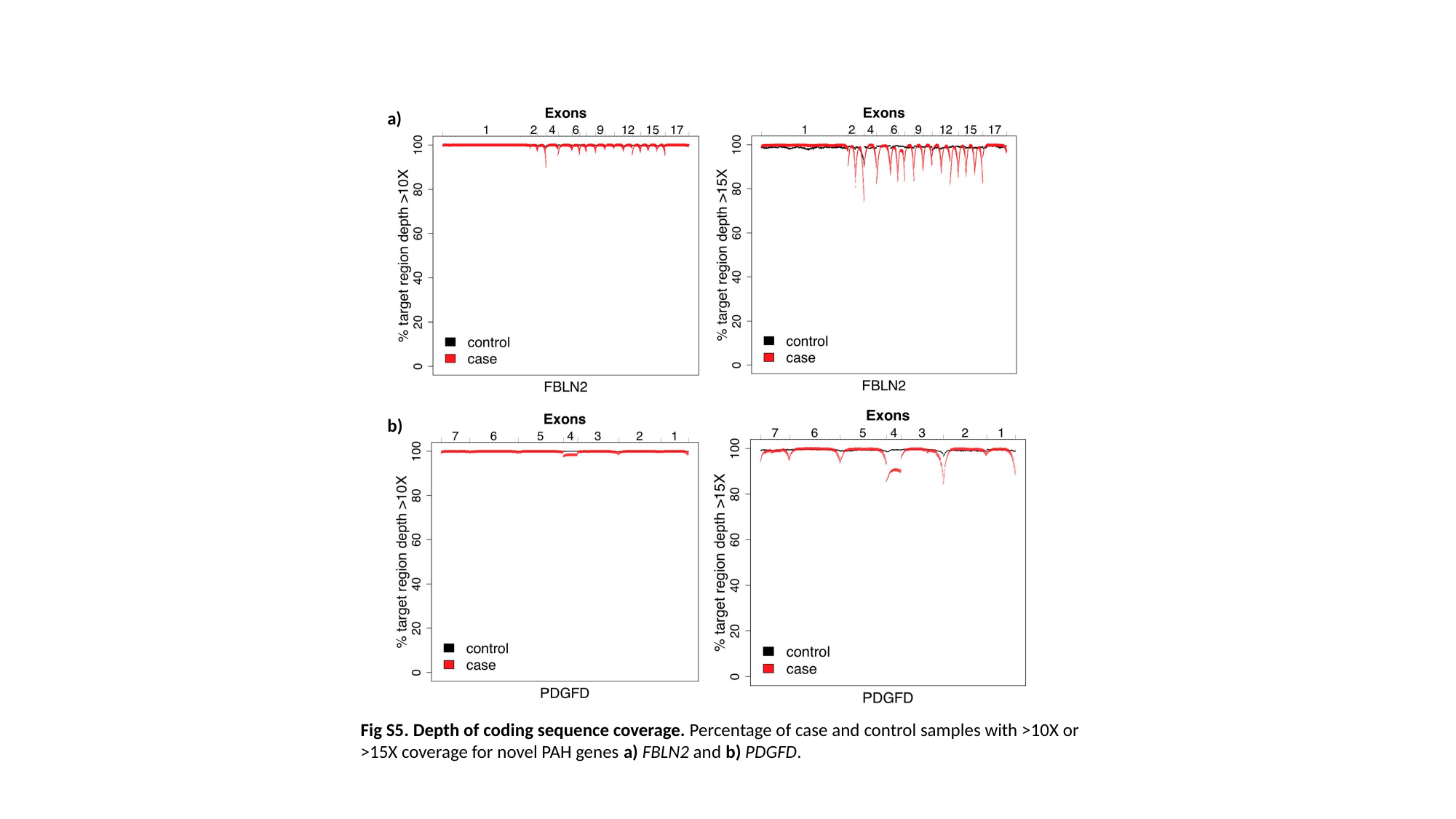

a)
b)
Fig S5. Depth of coding sequence coverage. Percentage of case and control samples with >10X or >15X coverage for novel PAH genes a) FBLN2 and b) PDGFD.

## Slide 6
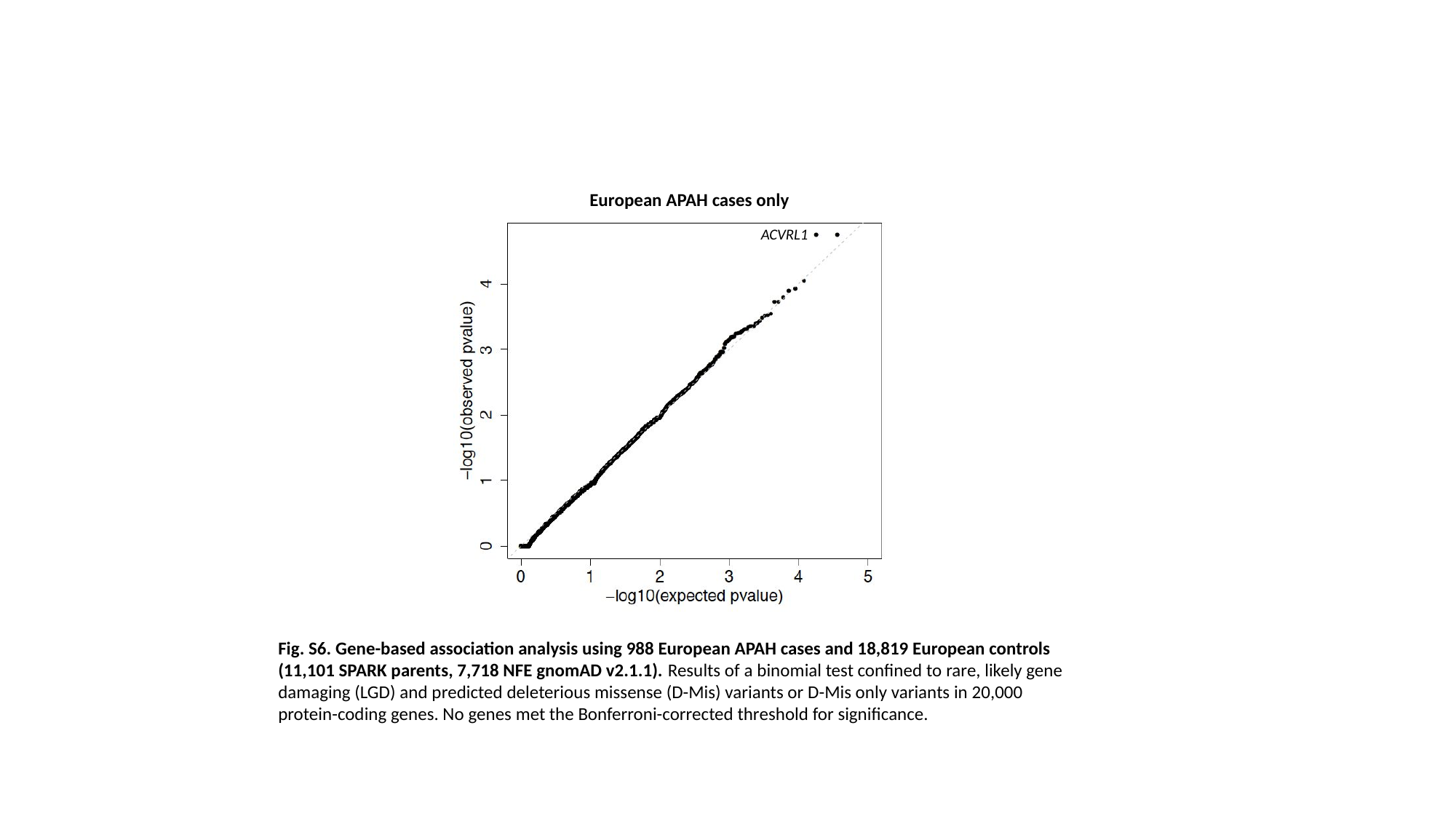

European APAH cases only
ACVRL1
Fig. S6. Gene-based association analysis using 988 European APAH cases and 18,819 European controls (11,101 SPARK parents, 7,718 NFE gnomAD v2.1.1). Results of a binomial test confined to rare, likely gene damaging (LGD) and predicted deleterious missense (D-Mis) variants or D-Mis only variants in 20,000 protein-coding genes. No genes met the Bonferroni-corrected threshold for significance.
