# Supplementary material for: Rare variant analysis of 4241 pulmonary arterial hypertension cases from an international consortium implicates FBLN2, PDGFD, and rare de novo variants in PAH
Source: Genome Med. 2021 May 10;13:80. doi: 10.1186/s13073-021-00891-1 (PMC8112021; doi:10.1186/s13073-021-00891-1)
Supplement: Supplementary file 2 — Additional file 2: Supplementary Table 1. Clinical characteristics and hemodynamic parameters of child- vs adult-onset PAH cases. Supplementary Table 2. Similar frequency of rare synonymous variants among cases and controls. Supplementary Table 3. Rare predicted deleterious KDR missense variants. Supplementary Table 4. Haplotype analysis of PAH cases with recurrent variants in new candidate genes. Supplementary Table 5. Burden of de novo variants in pediatric-onset IPAH. Supplementary Table 6. Rare de novo risk variants identified in pediatric-onset PAH. Supplementary Table 7. Clinical characteristics of pediatric PAH cases with rare de novo variants. [file 13073_2021_891_MOESM2_ESM.docx]

**Table S1. Clinical characteristics and hemodynamic parameters of child- vs adult-onset PAH cases* at diagnosis.**

| **Group** | **Age at dx**  **(y)** | **F:M ratio** | **MPAP**  **(mm Hg)** | **MCWP**  **(mm Hg)** | **CO, Fisk (L/min)** | **PVR**  **(Woods units)** |
| --- | --- | --- | --- | --- | --- | --- |
| Child (n=226) | 7.7 ± 5.4  (226) | 1.65:1 | 55.1 ± 18.6 (225) | 9.0 ± 3.0  (220) | 3.2 ± 1.6 (168) | 18.1 ± 11.7 (168) |
| Adult (n=2345) | 51.6 ± 14.7 (2345) | 4.02:1 | 49.6 ± 13.9 (2293) | 10.2 ± 4.2  (2231) | 4.6 ± 1.7 (1630) | 10.0 ± 5.9 (1630) |
| P-value | <0.0001** | <0.0001*** | <0.0001** | <0.0001** | <0.0001** | <0.0001** |

*Data are from the PAH Biobank (n=2,572 cases). Child-onset, <18 years of age at diagnosis.

Abbreviations: dx, diagnosis; F:M, female:male; MPAP, mean pulmonary artery pressure; MCWP, mean capillary wedge pressure; CO, cardiac output; PVR, pulmonary vascular resistance.

**Student’s t-test, 2-tailed

***Fisher’s exact test, 2-tailed

**Table S2. Similar frequency of rare synonymous variants among European PAH cases and controls.**

| **Mutation type*** | **PAH cases**  **(n = 2789)** | **Controls**  **(n = 18819)** | **Enrichment** | **P-value** |
| --- | --- | --- | --- | --- |
| SYN, all | 116982 | 792028 | 1.0 | 0.28 |
| SYN, predicted cryptic splice site | 795 | 5652 | 0.95 | 0.18 |
| MIS | 236401 | 1598016 | 1.0 | 0.41 |
| Indel | 12898 | 88577 | 0.98 | 0.06 |

*SYN, synonymous; MIS, missense; Indel, insertion/deletion.

**Table S3. Rare predicted deleterious *KDR* missense variants* among 4,175 PAH cases**.**

| **Case ID** | **Sex** | **Age_dx_** | **PAH subclass** | **Ancestry** | **Gene ***** | **Exon** | **Nucleotide change** | **Amino acid change** | **Variant type** | **MAF (gnomAD exomes)** | **CADD score** | **Revel** |
| --- | --- | --- | --- | --- | --- | --- | --- | --- | --- | --- | --- | --- |
| 06-049 | F | 53 | APAH-CHD | EUR | *KDR* | 23 | c.3089C>G | p.(Ala1030Gly) | D-Mis | --- | 29.3 | 0.87 |
| 22-037 | F | 43 | IPAH | EUR | *KDR* | 23 | c.3175T>C | p.(Tyr1059His) | D-Mis | --- | 29.4 | 0.90 |
| E011155 | F | 74 | IPAH | EUR | *KDR* | 25 | c.3311C>A | p.(Ser1104Tyr) | D-Mis | 3.61E-05 | 29.5 | 0.86 |
| 15-032 | M | 25 | IPAH | EUR | *KDR* | 26 | c.3439C>T | p.(Pro1147Ser) | D-Mis | 9.16E-05 | 25.2 | 0.88 |
| E013241 | F | 73 | IPAH | EUR | *KDR* | 26 | c.3439C>T | p.(Pro1147Ser) | D-Mis | 9.16E-05 | 25.2 | 0.88 |
| W000314 | M | 75 | IPAH | EUR | *KDR* | 26 | c.3439C>T | p.(Pro1147Ser) | D-Mis | 9.16E-05 | 25.2 | 0.88 |

*Rare, deleterious variants defined as gnomAD_exome_ALL AF ≤1.00E-04 and LGD or missense with variable REVEL cut-off (*KDR* 0.86).

**Cases are heterozygous for the indicated variants.

***Transcript: *KDR* NM_002253.3

**Table S4. Characteristics of longest shared haplotypes among PAH cases with recurrent variants in new candidate risk genes shows that the haplotype lengths are small and therefore do not support a common ancestor.** Genomic position are data based on human assembly GRCh38/hg38.

| Gene | Variant position | Haplotype position | Haplotype length | Variant carrier IDs | Frequency in EUR cases | Frequency in EUR controls |
| --- | --- | --- | --- | --- | --- | --- |
| *KDR* | 4:55088939 | 4:55079914-55088939 | 9025 | 15-032 E013241 W000314 | 2767/2790 | 10996/11088 |
| *KDR* | 4:55092622 | 4:55080187-55092622 | 12435 | W000274 27-015 | 2247/2790 | 8958/11088 |
| *FBLN2* | 3:13630815 | 3:13630900-13613921 | 16979 | 23-001 29-031 34-005 W000210 | 1367/2790 | 5624/11088 |
| *PDGFD* | 11:103926920 | 11:103909727-104000027 | 90300 | E000820 E010173 | 1585/2790 | 5703/11088 |
| *PDGFD* | 11:103996172 | 11:103909727-104038007 | 128280 | E012465 E014342 | 129/2790 | 501/11088 |

**STable S5. Burden of *de novo* variants in pediatric-onset IPAH (n = 66 child-parent trios).**

|  | **Variant type*** | **Observed** | **Expected by chance** | **Enrichment** | **P-value** | **Estimated # of risk variants** |
| --- | --- | --- | --- | --- | --- | --- |
| All genes  (18939 genes) | SYN | 18 | 20.4 | 0.88 | 0.74 | N/A |
|  | LGD | 9 | 6.3 | 1.43 | 0.31 | N/A |
|  | MIS | 53 | 45.1 | 1.18 | 0.23 | N/A |
|  | D-Mis | 17 | 9.2 | 1.84 | 0.019 | N/A |
|  | LGD + D-Mis | 26 | 15.4 | 1.69 | 0.014 | 11 |
| HLE or HHE**  (5756 genes) | SYN | 10 | 7.5 | 1.34 | 0.35 | N/A |
|  | LGD | 6 | 2.5 | **2.4** | **0.04** | N/A |
|  | MIS | 23 | 16.9 | 1.36 | 0.14 | N/A |
|  | D-Mis | 11 | 3.9 | **2.85** | **0.002** | **6** |
|  | LGD + D-Mis | 17 | 6.3 | **2.7** | **0.0003** | **11** |

* SYN, synonymous; LGD, likely gene-disrupting; MIS, missense; D-Mis, deleterious missense based on REVEL >0.5.

**HLE, high lung expression (murine E16.5 lung stromal cells); HHE, high heart expression (murine E14.5 heart).

**Table S6**. **Rare *de novo* LGD or D-Mis risk variants identified in 124 pediatric-onset PAH trios.**

| **Gene symbol** | **Gene name** | **Transcript** | **Variant type** | **Nucleotide change** | **Protein change** | **REVEL score** | **AF gnomAD exomes** | **pLI** | **Lung expression (% rank)** | **Heart expression**  **(% rank)** | **Gene-level associated medical condition(s)**  **(OMIM #, mode of inheritance)** |
| --- | --- | --- | --- | --- | --- | --- | --- | --- | --- | --- | --- |
| *ACVRL1* | Activin A receptor, type II like 1 | NM_001077401.2 | D-Mis | c.955G>C | p.(Gly319Arg) | 0.83 | . | 0.01 | 88.70 | 51.42 | Hereditary hemorrhagic telangiectasia  (600376, AD) |
| *ALDH9A1* | Aldehyde dehydrogenase family 9 subfamily A member 1 | NM_000696.3 | D-Mis | c.545A>G | p.(Tyr182Cys) | 0.88 | 2.44E-05 | 0.00 | 57.11 | 59.25 |  |
| *AMOT* | Angiomotin | NM_001113490.1 | LGD | c.957delC | p.(.Leu320Cysfs*55) | . | . | 0.21 | 67.96 | 94.51 |  |
| *ATP6V0A2* | ATPase, H+ transporting, lysosomal, VO subunit A2 | NM_012463.4 | D-Mis | c.1184A>G | p.(Asn395Ser) | 0.82 | . | 0.00 | 70.43 | 61.38 |  |
| *BMPR2* | Bone morphogenetic protein receptor, type II | NM_001204.7 | D-Mis | c.1471C>T | p.(Arg491Trp) | . | . | 1.00 | 86.44 | 42.01 | PAH (178600, AD)  PVOD (265450, AD) |
| *BMPR2* | Bone morphogenetic protein receptor, type II | NM_001204.7 | LGD | c.418+1G>A | p.(=) | 0.96 | . | 1.00 | 86.44 | 42.01 | PAH (178600, AD)  PVOD (265450, AD) |
| *BRWD3* | Bromodomain- and WD repeat-containing protein 3 | NM_153252.5 | D-Mis | c.1087G>T | p.(Asp363Tyr) | 0.58 | . | 1.00 | 61.56 | 47.34 | Mental retardation (300659, XLR) |
| *CHRNA4* | Cholinergic receptor, neuronal nicotinic, alpha polypeptide 4 | NM_000744.7 | D-Mis | c.721C>T | p.(Arg241Trp) | 0.92 | . | 0.02 | 29.90 | 18.42 | Epilepsy (600513, AD) |
| *CNTN4* | Contactin 4 | NM_001206956.1 | D-Mis | c.722A>G | p.(His241Arg) | 0.66 | . | 1.00 | 42.65 | 23.58 |  |
| *CSNK2A2* | Casein kinase II, alpha 2 | NM_001896.4 | D-Mis | c.551A>T | p.(His184Leu) | 0.50 | . | 1.00 | 54.88 | 77.13 |  |
| *DNMT3A* | DNA methyltransferase 3A | NM_153759.3 | D-Mis | c.473T>C | p.(Leu158Pro) | 0.78 | . | 0.00 | 98.27 | 96.73 | Acute myeloid leukemia (601626)  Heyn-Sproul_Jackson syndrome (618724, AD), Tatton-Brown-Rahman syndrome (615879, AD) |
| *EMC8* | Endoplasmic reticulum membrane protein complex subunit 8 | NM_006067.5 | LGD | c.633G>C | p.(*211Tyrext*15) | . | 4.06E-06 | 0.26 | 72.18 | 66.27 |  |
| *EMID1* | Emi domain-containing protein 1 | NM_001267895.2 | D-Mis | c.1114G>A | p.(Gly372Arg) | 0.77 | . | 0.04 | 55.13 | 36.41 |  |
| *GAMT* | Guanidinoacetate methyltransferase | NM_000156.6 | D-Mis | c.490G>T | p.(Gly164Cys) | 0.92 | . | 0.00 | 42.58 | 65.43 | Cerebral creatinine deficiency syndrome 2 (612736, AR) |
| *GDPD4* | Glycerophosphodiester phosphodiesterase domain containing 4 | NM_182833.3 | LGD | c.1561T>A | p.(*521Lysext*64) | . | . | 0.00 | 9.53 | 0.00 |  |
| *GRHL2* | Grainyhead-like transcription factor 2 | NM_001330593.2 | D-Mis | c.749C>T | p.(Pro250Leu) | 0.62 | . | 1.00 | 35.55 | 28.19 | Corneal dystrophy (618031, AD)  Deafness (608641, AD)  Ectodermal dysplasia/short stature syndrome (616029, AR) |
| *HNRNPF* | Heterogeneous nuclear ribonucleoprotein F | NM_001098208.1 | LGD | c.629delA | p.(Tyr210Leufs*14) | . | . | 0.86 | 85.44 | 97.61 |  |
| *HSPA4* | Heat shock protein family A (HSP70) member 4 | NM_002154.4 | D-Mis | c.2051C>G | p.(Pro684Arg) | 0.62 | 4.10E-06 | 0.03 | 42.59 | 95.61 |  |
| *ITPR1* | Inositol 1,4,5-triphosphate receptor type 1 | NM_001168272.1 | D-Mis | c.3614C>T | p.(Ala1205Val) | 0.69 | 1.69E-05 | 1.00 | 94.57 | 78.37 | Gillespie syndrome (206700, AD, AR)  Spinocerebellar ataxia (606658, AD; 117360, AD) |
| *KDM3B* | Lysine demethylase 3B | NM_016604.4 | D-Mis | c.3298C>T | p.(Pro1100Ser) | 0.66 | . | 1.00 | 89.37 | 86.59 |  |
| *KEAP1* | Kelch-like ECH-associated protein 1 | NM_012289.4 | LGD | c.1752C>A | p.(Tyr584*) | . | . | 0.25 | 79.29 | 82.09 |  |
| *MBTPS1* | Membrane-bound transcription factor protease, site 1 | NM_003791.4 | D-Mis | c.1342G>A | p.(Ala448Thr) | 0.61 | 5.41E-06 | 0.13 | 97.85 | 95.59 | Spondyloepiphyseal dysplasia  (618392, AR, 1 patient only) |
| *MECOM* | MDS1 and EVI1 complex locus | NM_001163999.1 | D-Mis | c.2285T>C | p.(Phe762Ser) | 0.76 | . | 1.00 | 81.79 | 59.65 | Radioulnar synostosis and amegakaryocytic thrombopenia (RUSAT2, 616738, AD) |
| *MFN2* | Mitofusin 2 | NM_001127660.1 | D-Mis | c.311G>A | p.(Arg104Gln) | 0.94 | . | 1.00 | 76.46 | 96.94 | Charcot-Marie-Tooth disease (609260, AD; 617087, AR)  Hereditary motor and sensory neuropathy (601152, AD) |
| *MYOM1* | Myomesin 1 | NM_019856.2 | LGD | c.3019C>T | p.(Arg1007*) | . | 1.27E-05 | 0.00 | 57.08 | 99.42 |  |
| *NOTCH1* | Notch receptor 1 | NM_017617.5 | D-Mis | c.1430T>A | p.(Ile477Asn) | 0.74 | . | 1.00 | 87.73 | 87.89 | Adams-Oliver syndrome (616028AD)  Aortic valve disease (109730, AD) |
| *NUCB1* | Nucleobindin 1 | NM_006184.6 | LGD | c.568dupT | p.(Tyr190Leufs*29) | . | . | 0.00 | 94.51 | 93.26 |  |
| *OLFML2B* | Olfactomedin-like 2B | NM_001297713.1 | D-Mis | c.1694T>A | p.(Ile565Asn) | 0.69 | . | 0.00 | 74.96 | 75.85 |  |
| *PSMD12* | Proeasome 26S subunit, non-ATPase 12 | NM_174871.4 | LGD | c.1207_1209del | p.(Asn403del) | . | . | 1.00 | 84.88 | 89.07 | Stankiewicz-Isidor syndrome (617516, AD) |
| *PTPN11* | Protein tyrosine phosphatase non-receptor type 11 | NM_002834.5 | D-Mis | c.182A>G | p.(Asp61Gly) | 0.92 | . | 1.00 | 92.32 | 94.20 | LEOPARD syndrome (151100, AD)  Leukemia (607785)  Metachondromatosis (156250, AD)  Noonan syndrome (163950, AD) |
| *PTPRK* | Protein tyrosine phosphatase receptor type K | NM_001291984.2 | D-Mis | c.4202T>C | p.(Val1401Ala) | 0.54 | . | 0.98 | 93.06 | 90.50 |  |
| *RAF1* | Raf-1 proto-oncogene, serine/threonine kinase | NM_002880.3 | D-Mis | c.781C>A | p.(Pro261Thr) | 0.87 | . | 1.00 | 26.87 | 91.43 | Cardiomyopathy (615916, AD)  LEOPARD syndrome (611554)  Noonan syndrome (611553, AD) |
| *RASA2* | RAS P21 protein activator 2 | NM_001303245.2 | D-Mis | c.1916C>T | p.(Thr639Ile) | 0.56 | 1.63E-05 | 0.00 | 74.96 | 66.34 |  |
| *SLC25A24* | Solute carrier family 25 member 24 | NM_013386.5 | D-Mis | c.649C>T | p.(Arg217Cys) | 0.81 | . | 0.00 | 77.16 | 73.97 | Fontaine progeroid syndrome (612289, AD) |
| *SLC38A6* | Solute carrier family 38 member 6 | NM_153811 | LGD | c.364-1G>T | p.(=) | . | . | 0.00 | 44.38 | 33.57 |  |
| *SRPRA* | SPR receptor subunit alpha | NM_001177842.1 | D-Mis | c.100C>A | p.(Arg34Ser) | 0.64 | . | NA | NA | NA |  |
| *TBX4* | T-box transcription factor 4 | NM_018488.3 | LGD | c.293C>G | p.(Pro98Arg) | 0.97 | . | 0.41 | 98.98 | 24.64 | Ischiocoxopodopatellar syndrome with or without PAH (147891, AD)  Posterior amelia with pelvic and pulmonary hypoplasia syndrome (601360, AR) |
| *TBX4* | T-box transcription factor 4 | NM_018488.3 | LGD | c.538_547del | p.(Pro180Ilefs*45) | . | . | 0.41 | 98.98 | 24.64 | Ischiocoxopodopatellar syndrome with or without PAH (147891, AD)  Posterior amelia with pelvic and pulmonary hypoplasia syndrome (601360, AR) |
| *TBX4* | T-box transcription factor 4 | NM_018488.3 | D-Mis | c.985G>T | p.(Asp329Tyr) | 0.61 | . | 0.41 | 98.98 | 24.64 | Ischiocoxopodopatellar syndrome with or without PAH (147891, AD)  Posterior amelia with pelvic and pulmonary hypoplasia syndrome (601360, AR) |
| *TBX4* | T-box transcription factor 4 | NM_018488.3 | LGD | c.1054C>T | p.(Arg352*) | . | . | 0.41 | 98.98 | 24.64 | Ischiocoxopodopatellar syndrome with or without PAH (147891, AD)  Posterior amelia with pelvic and pulmonary hypoplasia syndrome (601360, AR)Posterior amelia with pelvic and pulmonary hypoplasia syndrome(601360) |
| *TRH* | Thyrotropin-releasing hormone | NM_007117.5 | D-Mis | c.253C>A | p.(His85Asn) | 0.54 | . | 0.00 | 12.25 | 75.66 | Thyrotropin-releasing hormone deficiency (275120, AR) |
| *TUBB6* | Tubulin beta 6 class V | NM_001303527.2 | DMis | c.40C>T | p.(Arg14Trp) | 0.68 | 8.13E-06 | 0.00 | 92.18 | 89.57 | Congenital facial palsy with ptosis and velopharyngeal dysfunction (617732, AD) |
| *ZMYM2* | Zinc finger MYM-type containing 2 | NM_001190965.3 | LGD | c.1618C>T | p.(Arg540*) | . | . | 0.97 | 93.10 | 77.22 |  |
| *ZNF620* | Zinc finger protein 620 | NM_175888.4 | LGD | c.74G>A | p.(Trp25*) | . | . | 0.00 | NA | NA |  |

*Rare, deleterious variants defined as gnomAD AF ≤1.00E-04 and LGD or missense with REVEL <0.5.

**Table S7. Clinical characteristics of pediatric PAH cases with rare *de novo* LGD or D-Mis variants.**

| **Gene symbol** | **Variant type** | **Gene-level associated medical condition(s)** | **Case ID** | **Sex** | **Age of dx** | **Genetic ancestry** | **PAH**  **class** | **PAH subclass** | **Heart defect** | **Growth & development phenotype** | **Other medical condition(s)** | **MPAP (mmHg)** | **MPCWP (mmHg)** | **CO Fick**  **(L/min)** | **PVR**  **(Woods units)** | **Vital status** |
| --- | --- | --- | --- | --- | --- | --- | --- | --- | --- | --- | --- | --- | --- | --- | --- | --- |
| *ACVRL1* | D-Mis | HHT | JM0057 | M | 6 | AFR | APAH | HHT |  |  | NA | 54 | 4 | 2.7 | 18.5 | Deceased |
| *ALDH9A1, TUBB6* | D-Mis | *TUBB6*: Congenital facial palsy with ptosis and velopharyngeal dysfunction | 15-002* | F | 9 | AFR | IPAH |  |  |  |  | 75 | 8 | 5.8 | 11.55 | Transfer to adult care |
| *AMOT, ZNF620* | LGD |  | JM0004* | M | 1 | EUR | IPAH |  |  |  |  | 36 | 11 | 3.6 | 6.94 | Alive 2016 |
| *ATP6V0A2* | D-Mis |  | JM1344 | M | 12.5 | EAS | IPAH |  |  | Autism |  | 54 | 9 | 2.4 | 12.08 | Alive 2020 |
| *BMPR2* | D-Mis | PAH; PVOD | JM625 | M | 3 | EUR | IPAH |  |  |  | Anemia, depression, renal failure | NA | NA | NA | NA | NA |
| *BMPR2*, *MECOM* | LGD | *BMPR2*: PAH; PVOD  *MECOM*: RUSAT2 | LP2001061* | F | 3 | EUR | IPAH |  |  |  |  | NA | NA | NA | NA | NA |
| *BRWD3, GDPG4* | D-Mis,  LGD | *BRWD3*: Mental retardation | JM140* | F | 3 | EUR | APAH | CHD | TOGV |  | Asthma, depression | 59 | 14 | 6.8 | 6.62 | Deceased (17 y) |
| *CHRNA4* | D-Mis | Epilepsy | JM187 | M | 0.75 | EUR | APAH | CHD | VSD, coarctation of the aorta | Multiple congenital anomalies |  | NA | NA | NA | NA | NA |
| *CNTN4* | D-Mis |  | JM0035 | F | 12 | EUR | IPAH |  |  |  |  | 29 | 5 | 3.8 | 6.32 | Deceased following tx |
| *CSNK2A2, SLC38A6,*  *TRH* | D-Mis,  LGD,  D-Mis | *TRH*: TRH deficiency | JM0028* | F | 9 | EUR | IPAH |  |  |  |  | 37 | 8 | 2 | 14.5 | Deceased |
| *DNMT3A* | D-Mis | Heyn-Sproul_Jackson syndrome, Tatton-Brown-Rahman syndrome, AML | JM217 | F | 15 | EUR | APAH | CHD | VSD, Eisenmenger syndrome | Down syndrome | Asthma, thyroid cancer | NA | NA | NA | NA | NA |
| *EMC8* | LGD |  | JM1307 | M | 3 | SAS | IPAH |  |  | NA | NA | NA | NA | NA | NA | NA |
| *EMID1* | D-Mis |  | 15-054 | M | 4 | AFR | IPAH |  |  |  |  | 34 | 9 | NA | NA | NA |
| *GAMT, MFN2* | D-Mis | *GAMT*: Cerebral creatinine deficiency syndrome 2  *MFN*: Charcot-Marie-Tooth disease,  Hereditary motor and sensory neuropathy | FPPH4004* | M | 10 | EUR | FPAH |  |  | NA | NA | NA | NA | NA | NA | NA |
| *GRHL2* | D-Mis | Corneal dystrophy,  Deafness, Ectodermal dysplasia/short stature syndrome | LP2000991 | M | 2 | EUR | IPAH |  | secundum ASD |  |  | 69 | NA | NA | NA | NA |
| *HNRNPF* | LGD |  | FPPH5703 | F | 0.25 | EUR | FPAH |  |  | PPHN, myelodysplastic syndrome | Microangiopathic hemolytic anemia, thrombocytopenia | 56 | 3 | 1.4 | 37.86 | Deceased (1.25 y) |
| *HSPA4* | D-Mis |  | JM192 | F | 15 | EUR | APAH | CHD | ASD, PDA |  |  | NA | 6 | 12.9 | 0.93 | NA |
| *ITPR1* | D-Mis |  | 15-042 | M | 5 | EUR | IPAH |  |  |  |  | 106 | 5 | 5.6 | 18.04 | Alive 2018 |
| *KDM3B* | D-Mis |  | 15-006 | F | 8 | EUR | IPAH |  |  |  |  | 37 | 5 | NA | NA | Alive 2018 |
| *KEAP1* | LGD |  | JM852 | F | 2 | EUR | IPAH |  |  | Developmental delay, Incontinentia pigmenti, spastic diplegia |  | 63 | 11 | 1.5 | 34.67 | Deceased |
| *MBTPS1* | D-Mis | Spondyloepiphyseal dysplasia (1 patient) | JM0024 | F | 4 | EUR | APAH | CHD | ASD, dextrocardia | Small stature for age |  | 52 | 9 | 2.2 | 19.55 | Alive 2020 |
| *MYOM1* | LGD |  | JM1367 | F | 8 | EUR | IPAH |  | Secundum ASD | Chronic lung disease of prematurity |  | NA | 7 | NA | NA | Alive 2020 |
| *NOTCH1* | D-Mis | Adams-Oliver syndrome  Aortic valve disease | JM1357 | F | 1 | EUR | APAH | CHD | TOF | Failure to thrive |  | 52 | 8 | NA | NA | Deceased (11 y) |
| *NUCB1* | LGD |  | JM171 | F | 5 | EUR | IPAH |  |  | NA | NA | NA | NA | NA | NA | NA |
| *OLFML2B, RAF1* | D-Mis | *RAF1*: Cardiomyopathy  LEOPARD syndrome  Noonan syndrome | JM1088* | F | 13 | EUR | IPAH |  |  | Failure to thrive | Myxomatous AV valve (neoplasm) | NA | NA | NA | NA | NA |
| *PSMD12* | LGD | Stankiewicz-Isidor syndrome | 06-095 | F | 1 | EUR | APAH | CHD | PDA |  |  | 65 | 5 | 2 | 30.00 | NA |
| *PTPN11* | D-Mis | LEOPARD syndrome  Leukemia  Metachondromatosis  Noonan syndrome | JM155 | F | 6 | EUR | APAH | CHD | ASD | Noonan syndrome |  | 46 | 8 | 3.7 | 10.27 | NA |
| *PTPRK* | D-Mis |  | JM200 | F | 2 | EUR | APAH | CHD | ASD, PDA, TOGV, VSD |  |  | 82 | 5 | 1.7 | 45.29 | Deceased (9 y) |
| *RASA2* | D-Mis |  | JM138 | F | 0.42 | AMR | APAH | CHD | PDA | Congenital diplegia, small stature for age |  | 51 | 8 | NA | NA | NA |
| *SLC25A24* | D-Mis | Fontaine progeroid syndrome | JM216 | F | 2 | EUR | IPAH |  |  |  | Cystic fibrosis | 39 | 11 | 3.1 | 9.03 | Alive 2020 |
| *SRPRA* | D-Mis |  | FPPH133-01 | F | 15 | EUR | FPPH | HHT |  | Autism | Obsessive compulsive disorder | 69 | 10 | 3.2 | 18.44 | Deceased |
| *TBX4* | D-Mis | Ischiocoxopodopatellar syndrome with or without PAH  Posterior amelia with pelvic and pulmonary hypoplasia syndrome | JM0002 | F | 2 | Unknown | APAH | CHD | NA |  |  |  |  |  |  |  |
| *TBX4* | D-MIS | Ischiocoxopodopatellar syndrome with or without PAH  Posterior amelia with pelvic and pulmonary hypoplasia syndrome | 01-008 | F | 7 | EUR | IPAH |  |  |  |  | 40 | 8 | 4.8 | 6.67 |  |
| *TBX4* | LGD | Ischiocoxopodopatellar syndrome with or without PAH  Posterior amelia with pelvic and pulmonary hypoplasia syndrome | FPPH9002 | M | 2 | EUR | FPAH | Portal |  | NA | NA | NA | NA | NA | NA | NA |
| *TBX4* | LGD | Ischiocoxopodopatellar syndrome with or without PAH  Posterior amelia with pelvic and pulmonary hypoplasia syndrome | JM847 | M | 1-day | EUR | APAH | CHD | Alveolar hypoplasia |  |  | 66 | 10 | NA | NA | NA |
| *ZMYM2* | LGD |  | JM630 | M | 3 | EUR | IPAH |  |  | Actelectasis, bilateral lung; traction bronchiectasis; rib irregularities, bilateral; idiopathic scoliosis |  | 61 | 13 | 2.8 | 17.14 | Alive 2020 |

Abbreviations: AML, acute myeloid leukemia; RUSAT2, Radioulnar synostosis and amegakaryocytic thrombopenia; TRH, thyroid hormone deficiency.

*Multiple *de novo* variants identified in patient 15-002 (*ALDH9A1*, *TUBB6*), patient LP2001061 (*BMPR2* and *MECOM*), patient FPPH4004 (*GAMT*, *MFN2*), patient JM0004 (*AMOT*, *ZNF620*), patient JM0010 (*MANEA*, *RALGAPA1*), patient JM0028 (*CSNK2A2*, *TRH*), patient JM140 (*BRWB3*, *GDPD4*), patient JM1088 (*OLFML2B*, *RAF1*).
